# β-arrestin-2 in PAR-1-biased signaling has a crucial role in endothelial function via PDGF-β in stroke

**DOI:** 10.1038/s41419-019-1375-x

**Published:** 2019-02-04

**Authors:** Hideaki Kanki, Tsutomu Sasaki, Shigenobu Matsumura, Satoru Yokawa, Toshiro Yukami, Munehisa Shimamura, Manabu Sakaguchi, Tadahide Furuno, Takahiro Suzuki, Hideki Mochizuki

**Affiliations:** 1 Department of Neurology, Graduate School of Medicine, Osaka University, Yamadaoka 2-2, Suita, Osaka, 565-0871 Japan; 2 Laboratory of Nutrition Chemistry, Division of Food Science and Biotechnology, Graduate School of Agriculture, Kyoto University, 611-0011 Kyoto, Japan; 3 School of Pharmacy, Aichi Gakuin University, 1-100 Kusumoto-cho, Chikusa-ku, Nagoya, 464-8650 Japan; 4 Department of Health, Development and Medicine, Osaka University Graduate School of Medicine, Yamadaoka 2-2, Suita, Osaka, 565-0871 Japan; 5 School of Dentistry, Aichi Gakuin University, 1-100 Kusumoto-cho, Chikusa-ku, Nagoya, 464-8650 Japan

## Abstract

Thrombin aggravates ischemic stroke and activated protein C (APC) has a neuroprotective effect. Both proteases interact with protease-activated receptor 1, which exhibits functional selectivity and leads to G-protein- and β-arrestin-mediated-biased signal transduction. We focused on the effect of β-arrestin in PAR-1-biased signaling on endothelial function after stroke or high-fat diet (HFD). Thrombin had a rapid disruptive effect on endothelial function, but APC had a slow protective effect. Paralleled by prolonged MAPK 42/44 signaling activation by APC via β-arrestin-2, a lower cleavage rate of PAR-1 for APC than thrombin was quantitatively visualized by bioluminescence video imaging. HFD-fed mice showed lower β-arrestin-2 levels and more severe ischemic injury. The expression of β-arrestin-2 in capillaries and PDGF-β secretion in HFD-fed mice were reduced in penumbra lesions. These results suggested that β-arrestin-2-MAPK-PDGF-β signaling enhanced protection of endothelial function and barrier integrity after stroke.

## Introduction

Blood–brain barrier (BBB) is crucial for the maintenance of homeostasis in the central nervous system and dysfunction of BBB occurs in neurological disorders. The breakdown of BBB leads to hemorrhagic transformation and aggravation of edema, subsequently worsening stroke^1^. Endothelial cells are directly related to BBB function^2^. A new drug directly potentiates BBB function is a promising novel drug and activated protein C (APC) is a potent agent^3^. APC in complex with endothelial protein C receptor (EPCR) is thought to be lead to barrier protection via activating β-arrestin-2 pathway^4^. Then, we focus on the protective effect of APC via β-arrestin-2 in endothelial cells under ischemic condition.

APC inhibited tissue plasminogen activator-mediated brain hemorrhage transformation after stroke interactions with protease-activated receptor 1 (PAR-1)^5^ and improved neurological findings^6^, but the mechanisms are not completely understood. However, thrombin aggravated ischemic stroke due to vascular permeability^7^. Despite their opposing effects, both APC and thrombin interact with PAR-1, a 7-transmembrane G-protein-coupled receptor (GPCR), on endothelial cells. The activation of PAR-1 by thrombin tends to promote cell death and barrier disruption, while PAR-1 activation by APC tends to promote cell protection and barrier protection, and this is referred to as the “thrombin paradox”. Anti-thrombin drugs are used for treating acute ischemic stroke^8^ and for preventing recurrence^9^. However, these anti-thrombin drugs rarely induce hemorrhage. Therefore, the elucidation of the mechanism for endothelial protection by APC via β-arrestin-2 under ischemic condition leads to the development of new drugs with less side effects of hemorrhage.

APC is thought to activate β-arrestin-2^4^. Previously, β-arrestin was shown to desensitize GPCRs, but recent studies have reported to activate signaling pathways independent of G proteins by β-arrestin^10^. This biased signaling has been a recent focus of research, and β-arrestin has a pivotal role^11^. β-arrestin-1 and β-arrestin-2 are expressed in many organs and cells and play important roles in various physiological processes^10^. In myocardial infarction, the protective role of β-arrestin-2 was reported^12,13^. However, roles of β-arrestin-2 in neurological disorders are unclear. We hypothesized that β-arrestin-2 is required for the barrier integrity and cell protection.

To evaluate this hypothesis, we examined the effects of APC or thrombin on endothelial function via β-arrestin-2-dependent pathway in PAR-1-biased signaling. Importantly, we used bioluminescence video imaging to visualize proteins on the surface of living cells^14–16^, using a fusion protein of *Gaussia* luciferase (GLase) and human PAR-1 (GLase-PAR1) for understanding the PAR-1-biased signaling.

Free fatty acid (FFA) levels are increased in obese subjects and independently associated with greater risks of cardiovascular events^17,18^. In particular, saturated palmitic acid (PA), a long chain saturated FA prevalent in the western diet, activate inflammatory signaling^19^. High fat diet (HFD) mice exhibit increases in body weight and cholesterol levels, and chronic inflammation^20,21^. HFD-fed rats showed elevated endogenous thrombin potential^22^. Therefore, we thought HFD mice as a model for PAR-1 biased signaling in vivo. Using these mice, we performed transient middle cerebral artery occlusion (MCAO) and evaluated BBB integrity.

The aim of this study was to elucidate the protective effect of APC via β-arrestin-2 in endothelial cells under ischemic conditions. Our results indicate HFD mice show more thrombin and less APC compared with normal chow diet (NCD) mice and HFD mice are a seemingly suitable model to reproduce PAR-1-biased signaling. We demonstrate that β-arrestin-2 in PAR-1-biased signaling has protective effects under ischemic condition and in HFD-induced obesity. By quantitative bioluminescence imaging using a fusion protein of GLase and PAR-1, we show a slower cleavage rate of PAR-1 by APC than by thrombin. The β-arrestin-2-MAPK 42/44-PDGF-β signaling induces enhanced protection of endothelial function and barrier integrity.

## Materials and methods

### Mice

Adult male C57BL6N (Charles River Laboratories Japan, Yokohama, Japan) mice were used this study. The experimental protocol was approved by the institutional animal care and use committee of Osaka University Graduate School of Medicine. Mice were provided a normal diet (NCD) (Oriental Yeast; MF) or HFD (Research Diets; D12492) from weeks 7 to 11.

### ELISA

Mice were fed with NCD or HFD and studied after 1 day, 3 days, 1 week, 2 weeks, and 4 weeks. Mice were killed and blood sampling was performed by right ventricular injection. Blood samples were centrifuged at 2000×*g* for 10 min and serum were collected. ELISA was performed using Mouse Thrombin simple step ELISA kit (ab230933; Abcam, Cambridge, UK) and Mouse APC ELISA Kit (E-EL-M2668; Elabscience, Maryland, USA). Absorbance was measured at 450 nm, and concentrations were calculated by referring to a standard curve.

### Transient focal ischemia

Mice fed a normal diet or a HFD were used at 11 weeks. Right MCAO was performed, as described previously^23^. The MCA was occluded for 1 h using a suture and then reperfused. Only mice with less than 30% of baseline control microperfusion during the first minute of occlusion was used in subsequent experiments.

### Determination of the neurological score and infarct volume

A neurological test was performed at 48 h after MCAO^24^. The spontaneous activity, symmetry of movements, symmetry of forelimbs, climbing wall of the wire cage, reaction to touch on either side of the trunk, and response to vibrissae touch were evaluated on a scale from 0 to 3 points. 2,3,5-Triphenyltetrazolium chloride (TTC) staining was used to evaluate infarct volume, as previously described^25^.

### In vivo model for analysis of BBB permeability

Mice were injected with 100 µL of 4 % Evans Blue (Sigma-Aldrich, St. Louis, MO, USA) 24 h after MCAO, intravenously. After 1 h, mice were perfused with 0.9% NaCl, and the brain was removed and separated into hemispheres ipsilateral and contralateral to the MCAO. Then, each hemisphere supplemented with 500 µL of foramide and transferred to a 55 °C heat block and incubated for 24 h to extract Evans Blue from tissues. The foramide/Evans Blue mixture was centrifuged to pellet any remaining tissue fragments and absorbance was measured at 610 nm; 500 µL of formamide was used as a blank and Evans Blue extravasated per gram tissue was determined.

### Measurement of CBF

Changes in surface cerebral blood flow (CBF) were determined using a laser speckle blood flow imaging system (Omegazone; Omegawave, Helsinki, Finland) to obtain high-resolution two-dimensional images, as previously reported^26^.

### Evaluation of microcirculation and endothelial dysfunction

Microcirculation was assessed, as described previously^23^. In brief, plasma was visualized using dichlorotriazinyl amino fluorescein (DTAF; excitation 489 nm, emission 515 nm; Sigma-Aldrich, Tokyo, Japan). At 48 h after the induction of ischemia, 100 µL of DTAF conjugated to serum was injected into the tail vein for 10 s. Thirty seconds after injection, each mouse was decapitated, and the brain was fixed in 4% paraformaldehyde for 24 h. Fifty micrometers of thick brain slices were prepared using a vibratome and examined under a fluorescence microscope.

### Immunofluorescence

The 50 µm thick brain sections were blocked with 10% normal donkey serum and incubated in the appropriate antibody. After 16 h, the sections were washed three times with Tris Buffered Saline with Tween 20 (0.05 M Tris-HCl, 0.15 M NaCl, 0.05% Tween 20) and the sections were incubated with Alexa 594-conjugated donkey anti-rabbit antibodies. DAPI was used to stain nuclei. Images were obtained by confocal microscopy.

### Cell culture and transfection

Bovine brain microvasculature endothelial cells (BBMCs) and human umbilical vein endothelial cells (HUVECs) were used. Cultures were grown for 24 h at 37 °C in a humidified atmosphere of 95% air and 5% CO_2_ before use in experiments. BBMCs were plated in a 10-cm dish coated with collagen in containing 10% fetal bovine serum (Invitrogen, Carlsbad, CA, USA) and 1% antibiotic-antimycotic (Invitrogen), and 10 ng/mL basic FGF (Roche Applied Science, Penzburg, Germany). HUVECs were also plated in a 10-cm dish coated with collagen in medium (Humedia-EG; Kurabo, Osaka, Japan).

An adenovirus-mediated reporter system was prepared. HUVECs were transfected with purified adenovirus, including miRNAs targeting β-arrestin-2 and control miRNA, and evaluated after 2 days. The miRNA sequence for the knockdown of β-arrestin-2 was 5′-TGCTGATACCTGGTCATCTTGTTCGAGTTTTGGCCACTGACTGACTCGAACAATGACCAGGTAT-3′. The sequence of control miRNA was LacZ-specific miRNAs. HUVECs samples were collected after 24 h in palmitic acid (WAKO) in 100% Ethanol conjugated to 10% bovine serum albumin.

### Oxygen-glucose deprivation

For oxygen-glucose deprivation (OGD)/reoxygenation, cells were washed with phosphate-buffered saline and incubated with glucose-free Earle’s balanced salts solution (Biological Industries Israel Beit Haemek Ltd., Beit Haemek, Israel) in an anaerobic chamber containing 5% CO_2_ and 95 % N_2_ (<1% O_2_) for 3 h, as previously reported^25^. After OGD, the medium was changed to the original medium and the cells were placed in a normoxic chamber for 18 h. Bovine thrombin (Sigma-Aldrich) and human APC (Haematologic Technologies, Essex Junction, VT, USA) were added immediately after OGD treatment.

### Tube-like capillary formation assay

The tube-like capillary formation assay was performed on Matrigel (Corning, Inc., Corning, NY, USA). Each well of 4-well culture plates, was coated with 100 µL of Matrigel, cells (1 × 10^5^ cells/mL) in reduced serum (5% FBS) were seeded, and drugs were added at the same time. The plate was incubated at 37 °C and after 18 h, tube-like capillary formation was observed. Tube length was determined by Image J 1.44 software (National Institutes of Health, Bethesda, MD, USA).

### Migration assay

The cells were plated on 10-cm dishes. After the reaching confluence, a wound was generated by scratching with 200-µL tips, followed by the addition of drugs. At 0 and 18 h after the scratch, the width of the wounds was determined using Image J.

### Western blotting

Cells on Matrigel were treated with APC, thrombin, forskolin (10 µM; WAKO, Osaka, Japan) or with OGD. After 18 h, cell lysates were obtained. Briefly, 400 µL of cell recovery solution (Corning BD # 35423) was added to chamber slides and Matrigel was scraped to make a Matrigel slurry followed by incubation on ice for 30 min to dissolve Matrigel. After centrifugation at 6000 rpm for 3 min at 4 °C, the pellet was resuspended in 100 µL of lysis buffer (20 mM/L Tri-HCl, 150 mM/L NaCl, 2 mM/EDTA, 1% Nonidet P-40, protease inhibitor cocktail (Complete Mini; Roche Applied Science) and phosphatase inhibitor cocktail (PhosSTOP; Roche Applied Science) and incubated on ice for 20 min. The supernatant was used for quantitation and further analysis. Cell samples were also collected in the lysis buffer and were centrifuged at 15,000 rpm for 20 min at 4 °C; the supernatant was used for western blotting. Samples were also isolated from the mouse cortex. The protein (10 µg) was subjected to electrophoresis in a 7.5% Tris-HCl polyacrylamide gel and transferred to a polyvinylidene difluoride membrane (Immobilon P; Millipore, Burlington, MA, USA). Blots were probed with the appropriate antibody and detected using a sheep anti-mouse or a donkey anti-rabbit HRP-conjugated secondary antibody (Amersham Pharmacia Biotech, Little Chalfont, UK) followed by enhanced chemiluminescence (ECL; Amersham Pharmacia Biotech). Digital images were obtained by densitometric scans of autoradiographs and quantified using Image J.

### Antibodies

Antibodies against β-arrestin-1 and β-arrestin-2 (Cell signaling, Danvers, MA, USA), PDGF-β (Abcam), β-actin (Sigma), MAPK 42/44 (Cell Signaling), and phosphorylated MAPK 42/44 (Cell Signaling) were used at a 1:1000 dilution for the western blot analysis and at a 1:500 dilution for immunofluorescence.

#### Plasmids

The fused protein of GLase and PAR-1 was used as a reporter protein to monitor PAR-1 cleavage activity by thrombin and APC. GLase-PAR1 is a fusion protein of human PAR-1, which has the peptide sequence of GLase (K18-R185 without the signal peptide sequence of GLase) between the signal peptide sequence of PAR-1 and the tethered-ligand sequence (Fig. 5a). As the cDNA encoding GLase, a preferred human codon-optimized *Gaussia* luciferase gene (*pGLuc*; JNC, Yokohama, Japan) was used^27,28^.

To express GLase-PAR1 (the fusion protein of wild-type PAR1) under the control of the cytomegalovirus (CMV) promoter, the pcDNA3-PAR1sp-pGLuc-PAR-1 vector was constructed as follows (Supplementary Fig. 4). The *Hin*dIII-*Eco*RI cDNA fragment encoding the human PAR-1 signal peptide sequence was obtained from the human PAR-1 cDNA IMAGE clone (3343051) by PCR amplification and inserted into pcDNA3-pGLuc-pNC to obtain pcDNA3-PAR1sp-pGLuc-pC. Next, the *Xho*I-*Xba*I cDNA fragment encoding human PAR-1 without the signal peptide sequence was obtained by PCR and inserted into pcDNA3-PAR1sp-pGLuc-pC to finally obtain pcDNA3-PAR1sp-pGLuc-PAR1.

To express GLase-PAR1 mutants with R41A, R46A, and R41A/R46A-double mutations in the PAR1 sequence, pcDNA3-PAR1sp-pGLuc-PAR1-R41A, -R46A, and -R41A/R46A were obtained using a PrimeSTAR Mutagenesis Kit (Takara Bio, Shiga, Japan). To express human EPCR, pcDNA3.2-EPCR-V5/DEST, or pcDNA6.2-Bsd/EmGFP-V5-DEST obtained by the Gateway system was used (Invitrogen).

To express the PDGF-β protein fused to the amino terminus of GLase (PDGF-β-GLase), pcDNA3-pPDGF-β-pGLuc was constructed as follows (Supplementary Fig. 7). A synthetic gene of the human PDGF-β preproprotein (*PDGF-β*) was obtained from Eurofins Genomics (Tokyo, Japan). The *Hind*III-*Eco*RI fragment of *PDGF-β* was amplified by PCR using the following primers: 5′-ccc AAGCTT AGCCACC ATG AAT CGC TGC TGG GCG CTC-3′ and 5′-ctt GAATTC GGC CCC GAG AGT CTC CTT GAG-3. The fragment was inserted into pcDNA3-pGLuc-pN^29^ to obtain pcDNA3-PDGF-β-pGLuc. To express GLase with its signal peptide sequence (GLsp; Supplementary Fig. 8C), pcDNA3-pGLuc was used^27^.

### Measurement of the luminescence activity of GLase using luminometers

The luminescence activity of GLase released from HEK293, CHO-K1, and mouse brain endothelial bEnd.3 cells expressing GLase-PAR1 after incubation with proteases was determined using luminometers. For the transient expression of GLase-PAR1 with or without EPCR, culture, and transfection conditions for various cells were as follows:

I. HEK293 cells: HEK293 cells (1 × 10^5^ cells) seeded in a 24-well plate (TPP) coated with collagen were pre-cultured for 48 h, and were transfected for 24 h or 48 h with 0.25 µg of the expression vector for GLase-PAR1 (pcDNA3-PAR1sp-pGLuc-PAR1) and 0.25 µg of the expression vector for EPCR (pcDNA3.2-EPCR-V5/DEST or the pcDNA3.2-V5/DEST empty vector for EPCR) using 1.5 µL of ViaFect transfection reagent (Promega) according to the manufacturer’s instructions.
II. Endothelial bEnd.3. cells: bEnd.3. cells (5 × 10^4^ cells) seeded in a 12-well plate (Falcon) coated with collagen were pre-cultured for 24 h and were transfected for 24 h with the expression vectors for GLase-PAR1 and EPCR (0.5 µg each) using 2 µL of Lipofectamine3000 transfection reagent (Invitrogen) with PLUS reagent (1 µL, Invitrogen) according to the manufacturer’s instructions.
III. CHO-K1 cells: CHO-K1 cells (1 × 10^5^ cells) seeded in a 24-well plate (TPP) coated with collagen were pre-cultured for 48 h, and were transfected for 72 h with the expression vectors for GLase-PAR1 (R41/R46 wild-type or R41A, R46A, or R41A/R46A-double mutants) and EPCR (0.25 µg each, respectively) using 2.5 µL of Lipofectamine 2000 transfection (Thermo Fisher Scientific, Waltham, MA, USA) reagent according to the manufacturer’s instructions.

After transfection, cells were washed three times with HBSS (Wako) and incubated for 30–60 min with HBSS containing 0.01% BSA with APC or thrombin at various concentrations. To estimate the amoun of GLase released from cells, the culture medium was collected by centrifugation at 300×*g* for 5 min at 4 °C to remove detached cells. To estimate the amount of GLase-PAR1 in cells, cells were lysed with Passive Lysis Buffer (Promega), and the cell lysate was obtained by centrifugation at 15,000×g for 10 min at 4 °C to remove insoluble proteins. The culture medium (1 µL) or cell lysate (1 µL) was used for measuring the luminescence activity of GLase by the addition of 50 µL of PBS containing 0.25 µg of coelenterazine. The initial maximal light intensity was measured at 0.1 s intervals for 5–10 s using an Atto (Tokyo, Japan) AB2200 luminometer (ver. 2.61D) in the presence of a neutral density filter or a Centro XS3 LB960 luminometer (Berthold Technologies, Bad Wildbad, Germany). Luminescence activity for each well is expressed as the mean values ± S.D.

The secreted PDGF-β-GLase from endothelial bEnd.3 cells were analyzed as follows. Endothelial bEnd.3 cells (1 × 10^5^ cells) seeded in a 24-well plate coated with collagen were pre-cultured for 48 h and transfected for 72 h with 0.25 µg of the expression vector for PDGF-β-GLase (pcDNA3-PDGF-β-pGLuc) and adeno-miRNA for β-arrestin-2 (or a control miRNA). To estimate the amount of PDGF-β-GLase secreted from endothelial bEnd.3 cells, the culture medium was collected by centrifugation at 300×g for 5 min at 4 °C to remove detached cells. To estimate the amount of PDGF-β-GLase in cells, cells were washed two times with PBS and lysed with 100 µL of Passive lysis buffer (Promega), and the cell lysate was obtained by centrifugation at 15,000×g for 10 min at 4 °C to remove insoluble proteins. The culture medium (1 µL) or cell lysate (1 µL) was used to measure the luminescence activity of GLase by the addition of 50 µL of PBS containing 0.25 µg of coelenterazine. The initial maximal light intensity was measured in 0.1 s intervals for 5–15 s using a Centro XS3 LB960 luminometer (Berthold). Luminescence activity in each well is expressed as the mean values ± S.D.

### Bioluminescence imaging

The method and system for video-rate bioluminescence imaging were essentially the same as those previously described^29,30^. Bioluminescence signals were monitored at 37 °C using a model IX81-ZDC2 microscope (Olympus, Tokyo, Japan) equipped with a thermostat incubator (Tokai Hit, Shizuoka, Japan) and water-cooled EM-CCD camera (ImagEM 1 K, model C9100-14, 1024 × 1024 pixels, pixel size = 13 µm; Hamamatsu Photonics, Hamamatsu, Japan) in a light-proof box. A high numerical aperture (NA) objective lens of UApo40×O (oil immersion lens; NA 1.35, Olympus) was used. Bioluminescence signals were recorded on a computer hard disk using AQUACOSMOS software version 2.6 (Hamamatsu Photonics) with an acquisition mode of 1 × 1 binning, fast scanning, EM gain level 255, and photon imaging model 1 in the software. The luminescence video images were processed and analyzed using the AQUACOSMOS software.

For live cell bioluminescence video imaging of GLase-PAR1 cleaved by APC and thrombin, transfection conditions were as follows:

i. CHO-KI cells (5 × 10^4^ cells) cultured for 24 h in a 35-mm glass-bottomed dish were transfected with 2 μg of the expression vectors (pcDNA3-PAR1sp-GLuc-PAR1: pcDNA3.2-EPCR = 1: 1) using 6 μL of ViaFect transfection reagent (Promega) and incubated for 48 h.
ii. HEK293 cells (1 × 10^5^ cells) cultured for 48 h on a 35-mm glass-bottomed dish were transfected with 2 μg of the expression vectors (pcDNA3-PAR1sp-GLuc-PAR1 only) using 6 µL of FuGENE HD (Promega) and incubated for 24 h.

Cells were washed three times with HBSS, and then the dish was supplemented with 0.5 mL of HBSS and set on a stage-top incubator on the microscope. The focus position of the *z*-axis was adjusted to 1 µm from the bottom of the cells attached to the glass plate using the ZDC autofocusing system. The luminescence reaction was started by the addition with 0.5 mL of pre-warmed HBSS containing 1 µg/mL coelenterazine, and the cells were pre-incubated for 10 min. During incubation, the focal planes were adjusted to luminescent cells of interest using the continuous autofocusing mode with the ZDC2 system to maintain the focus position. Following the further addition of 0.5 mL of pre-warmed HBSS containing 1 µg/mL coelenterazine with APC (3 µg/mL) or thrombin (0.05 or 0.5 U/mL), the luminescence video images of the cells were recorded for 20 min with an exposure time of 2 s and a reading time of 1.712 ms/image. The luminescence signals of the video images were converted to pseudo-colored images (Cyan). To analyze time-dependent changes in luminescence intensity for a video image, the average luminescence intensities in the defined cell areas were calculated.

### Transepithelial electrical resistance (TEER)

Transepithelial electrical resistance (TEER) was measured using a Millicell ERS Volt-ohm meter (Merckmillipore, Darmstadt, Germany). A 24-well dish and Cell Culture Inserts (Merckmillipore) coated with collagen at a density of 1 × 10^5^ per HUVEC cells/mL were used. Cultures were used for experiments 5 days after seeding and APC (0.3 ng/mL) or thrombin (0.5 U/ml) was added to the insert, then TEER was measured. After treatment with OGD, APC, or thrombin was added to the inserts, then TEER was measured.

### Permeability assay

Permeability was evaluated using Cell Culture Inserts (Merckmillipore). In the inserts, cells (1 × 10^5^ cells/insert) were seeded. After 5 days, APC (0.3 ng/mL) or thrombin (0.5 U/ml) was applied. After 3 h the medium of the insert was removed and phenol red-free medium containing lucifer yellow (1:1000, WAKO) was added, and absorbance was measured after 1 h at 562 nm.

### Angiogenesis array

The Proteome Profiler Mouse Angiogenesis Array Kit (R&D Systems) was used according to the manufacturer’s instructions to detect changes in the relative expression levels of 31 angiogeneis-related proteins in cells. Briefly, cell lysates were added to the antibody array membrane and incubated overnight. After incubation with HRP-conjugated streptavidin, signals were visualized by chemiluminescence.

### Quantitative real-time PCR

Levels of PDGF-α and -β were measured. RNA was isolated using the RNA Kit (ReliaPrep^TM^ RNA Cell Miniprep System; Promega) and cDNA was synthesized using the cDNA Kit (Transcription First Strand cDNA Synthesis Kit; Roche). Quantitative PCR was performed using SYBR Green PCR Master Mix (EXPRESS SYBR GreenER^TM^; Thermo Scientific) and the 7900 HT Real-time PCR system (Thermo Scientific). 36B4 was measured as an internal control using the 36B4 mRNA Control Kit. The primers for PDGF-α were 5′-CGTCCGCCAACTTCCTGAT-3′ and 5′-CCAAATGCTCCTCTAACCTCAC-3′ and the primer for PDGF-β were 5′-GCCTCATAGACCGCACCAAC-3′ and 5′-GCTTCTTCCGCACAATCTCG-3′. U0126 (10 µM; Sigma aldrich) and PD98059 (10 µm; Merckmillipore) were pretreated for 30 min and APC (1 ng/mL) was added in the medium and after 24 h, RNA was isolated.

### Statistical analysis

Almost all results are presented as means ± standard error of the mean or means ± standard deviation. Statistical analyses were performed using JMP 10.0.2 software (SAS Institute, Inc, Cary NC). Comparisons between groups were performed using Mann–Whitney *U*-tests. Comparison between more than two groups were analyzed using Kruskal–Wallis tests. Statistical significance was defined as *p* < 0.05.

## Results

### Potentiation of endothelial function by activated protein C (APC)

**Fig. 1:**
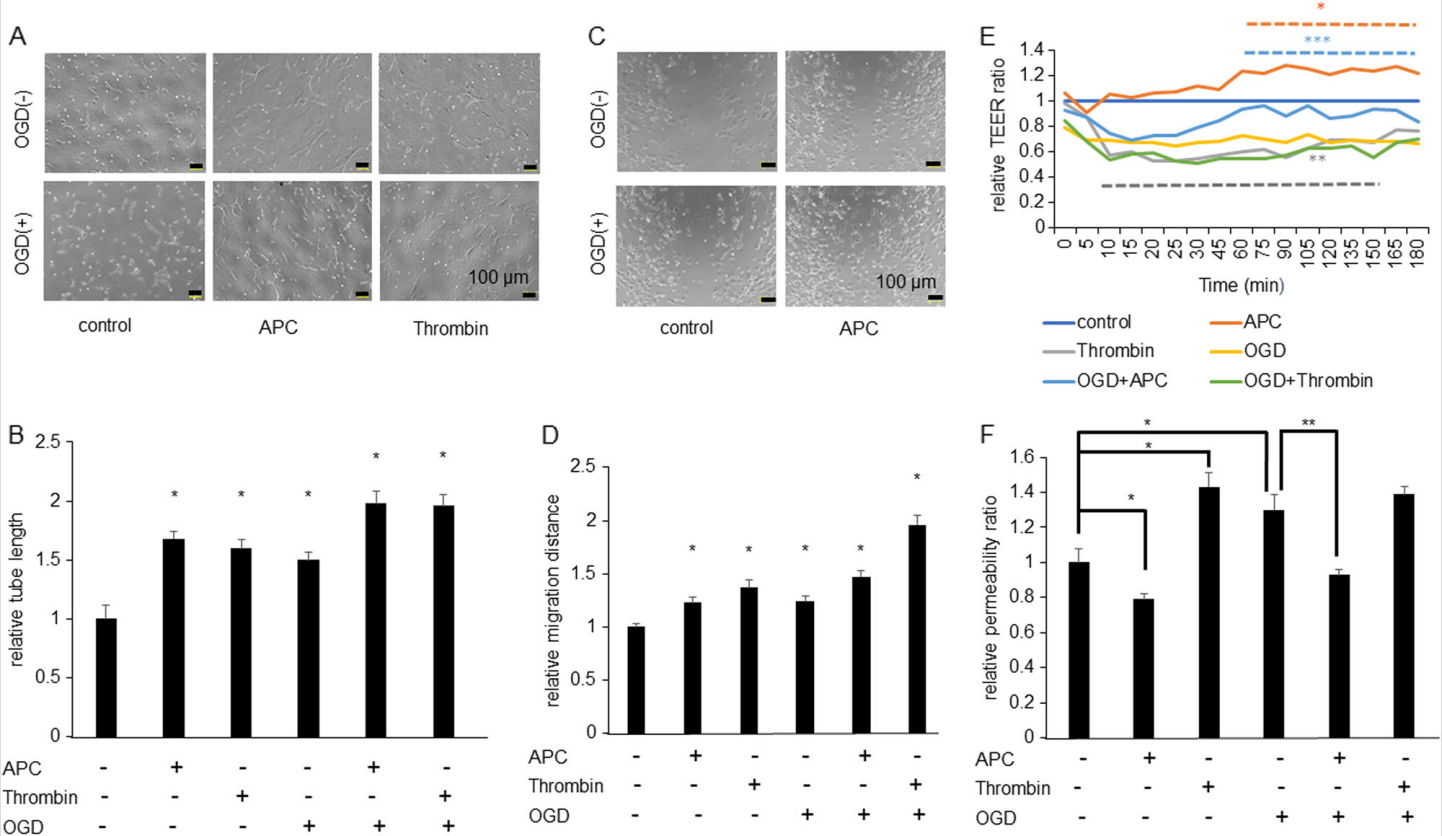
APC enhances tube-like capillary formation, migration, and transepithelial electrical resistance (TEER) and APC reduces permeability. **a** Tube-like capillary formation at Matrigel was examined. Upper panel showed representative image of BBMCs, BBMCs treated with thrombin 0.5 U/mL and BBMC treated with APC 0.3 ng/mL, lower panel showed each sample’s tube-like capillary formation after 18 h under treatment of OGD (1% O_2_ 3 h). Scale bar was 100 µm. **b** Quantitative results. Results are means ± SEM (*n* = 6). *p < 0.05 compared with the BBMC under no treatment by Mann–Whitney *U*-tests. **c** Representative migration assay image showed. Upper panel showed representative image of BBMCs under no treatment and after 18 h from the treatment with APC 0.3 ng/mL, and lower panel showed representative image of BBMCs after 18 h from OGD (1% O_2_ 3 h) treatment and OGD + APC 0.3 ng/mL treatment. Scale bar was 100 µm. **d** Quantitative results. Results are means ± SEM (*n* = 6). *p < 0.05 compared with the BBMCs under no treatment by Mann–Whitney *U*-tests. **e** Time course of TEER using HUVECs showed compared with HUVECs under no treatment (*n* = 5). * APC and ** thrombin, *p* < 0.05 compared with no treatment. *** APC + OGD, *p* < 0.05 compared with under ischemic condition (OGD) by Mann–Whitney *U*-tests. **f** Permeability assay showed compared with HUVECs under no treatment (*n* = 4 or 5). Results are means ± SEM (*n* = 4 or 5). Data were analyzed compared with multiple groups by Kruskal–Wallis tests. *p < 0.05 compared with HUVECs under no treatment and **p < 0.05 compared with HUVECs under ischemic condition (OGD)

We examined the effect of APC on endothelial function. Bovine brain microvasculature endothelial cells (BBMCs) and human umbilical vein endothelial cells (HUVECs) were subjected to OGD (oxygen-glucose deprivation: in vitro ischemia). APC showed significantly enhanced tube-like capillary formation at BBMCs on Matrigel (Fig. 1a, b). Thrombin and ischemia also potentiated tube-like capillary formation. APC, thrombin and ischemia significantly enhanced migration (Fig. 1c, d). These results showed that APC enhanced angiogenesis.

Thrombin leads to the decreased expression of occludin, claudin-5, and ZO-1 and rapidly induces vascular permeability^31,32^. Therefore, we examined the effect of APC on vascular permeability using transepithelial electrical resistance (TEER) assays. Thrombin reduced TEER rapidly and APC enhanced TEER more slowly (Fig. 1e). Ischemia also reduced TEER, but the effects of OGD were inhibited by APC.

A permeability assay also showed that thrombin enhanced paracellular permeability (Fig. 1f), while APC reduced paracellular permeability. Additionally, ischemia reduced permeability and APC mitigated the effect. These results showed that APC enhanced endothelial function under normal and ischemic conditions.

### Relationship between β-arrestin-2 in PAR-1-biased signaling and APC-induced angiogenesis

**Fig. 2:**
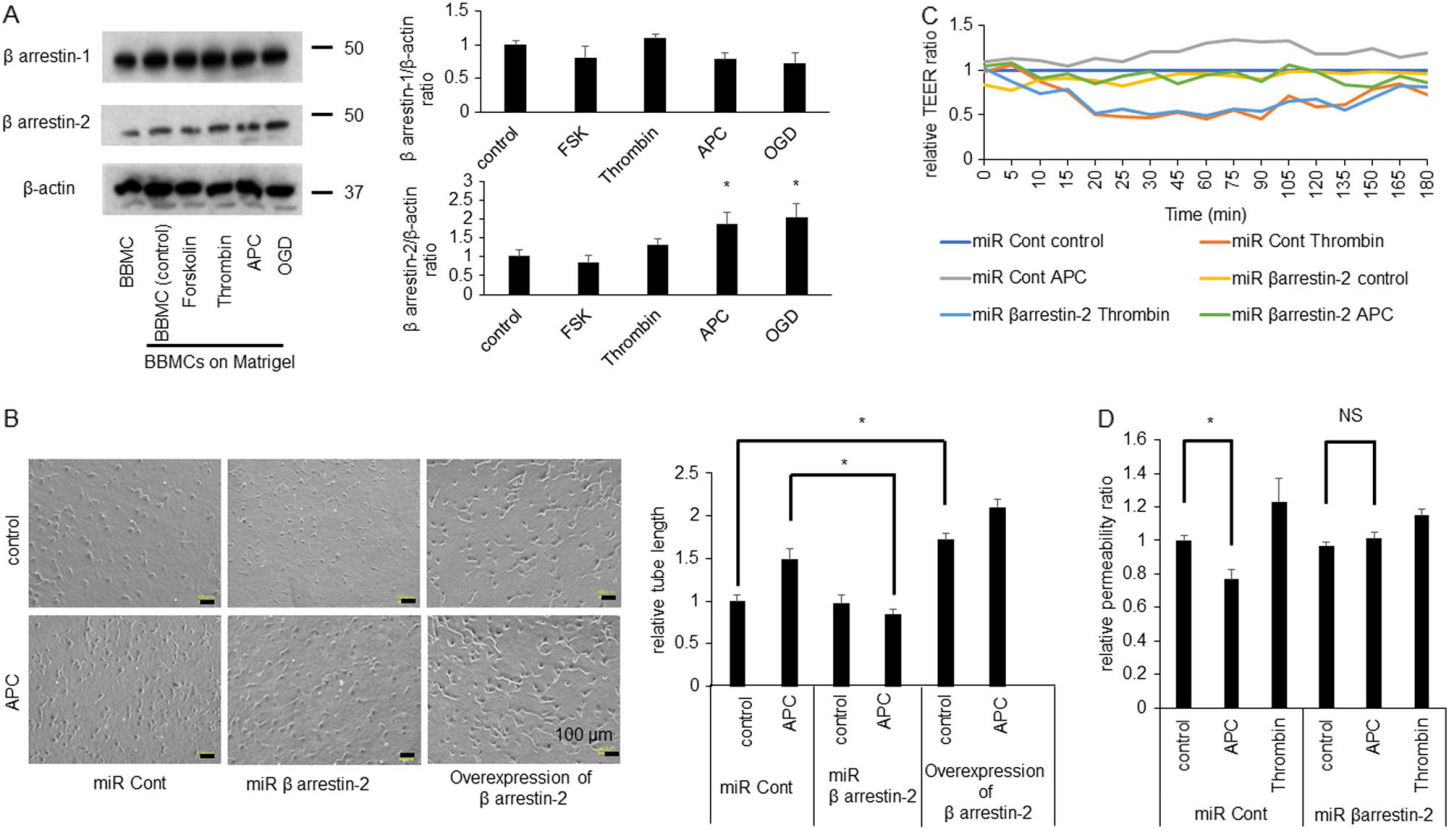
β-arrestin-2 is upregulated under treatment of APC and OGD. **a** Left panel showed western blot analysis using cell lysates from Matrigel. Right upper panel showed quantitative results of β-arrestin-1/β-actin ratio of western blot analysis (*n* = 4). Right lower panel showed quantitative results of β-arrestin-2/β-actin ratio of western blot analysis (*n* = 3–4). Data were analyzed compared with multiple groups by Kruskal–Wallis tests. *p < 0.05 compared with BBMCs on matrigel. **b** Left upper panel showed representative image of HUVECs using a miRNA control (miR Cont), knockdown of β-arrestin-2 by using miRNA (miR β arrestin-2) and overexpression of β-arrestin-2, left lower panel showed each sample tube-like capillary formation under treatment with APC (0.3 ng/mL). Scale bar was 100 µm. Right panel showed quantitative results. Results are means ± SEM (*n* = 6). Data were analyzed compared with multiple groups by Kruskal–Wallis tests. *p < 0.05. **c** Time course of TEER using knockdown of β-arrestin-2 HUVECs (miR βarrestin-2) compared with control HUVECs (miR Cont) showed (*n* = 4). **d** Permeability assay showed compared with control HUVECs (miR Cont) under no treatment (*n* = 6–9). Results are means ± SEM (*n* = 6–9). Data were analyzed compared with multiple groups by Kruskal–Wallis tests. *p < 0.05 compared with the HUVECs under no treatment. NS was meant not significantly changed

APC in complex with EPCR can also activate PAR-1^33-35^ and relate with β-arrestin-2^4^. To elucidate the mechanism underlying enhanced endothelial function by APC, we examined the β-arrestin-2-dependent pathway under treatment with APC or OGD. Western blot analysis showed expression levels of β-arrestin-2, but not β-arrestin-1, were increased in response to APC or OGD (Fig. 2a). Forskolin, which enhances endothelial function, did not affect expression levels of β-arrestin-1 or β-arrestin-2^36^. Then, we knocked down β-arrestin-2 (using the microRNA; miR β-arrestin-2) or overexpressed β-arrestin-2 using adenoviral-mediated gene transduction and compared the results with those obtained using a miRNA control (miR Cont; Supplementary Fig. 1A). The knockdown of β-arrestin-2 reduced tube-like capillary formation and the overexpression of β-arrestin-2 enhanced under APC treatment (Fig. 2b). The knockdown of β-arrestin-2 also reduced migration (Supplementary Fig. 1B), reduced TEER, and increased permeability (Fig. 2c, d). These results suggested that β-arrestin-2 has a pivotal role in angiogenesis and endothelial function.

### Slow and sustained MAPK 42/44 phosphorylation by APC

**Fig. 3:**
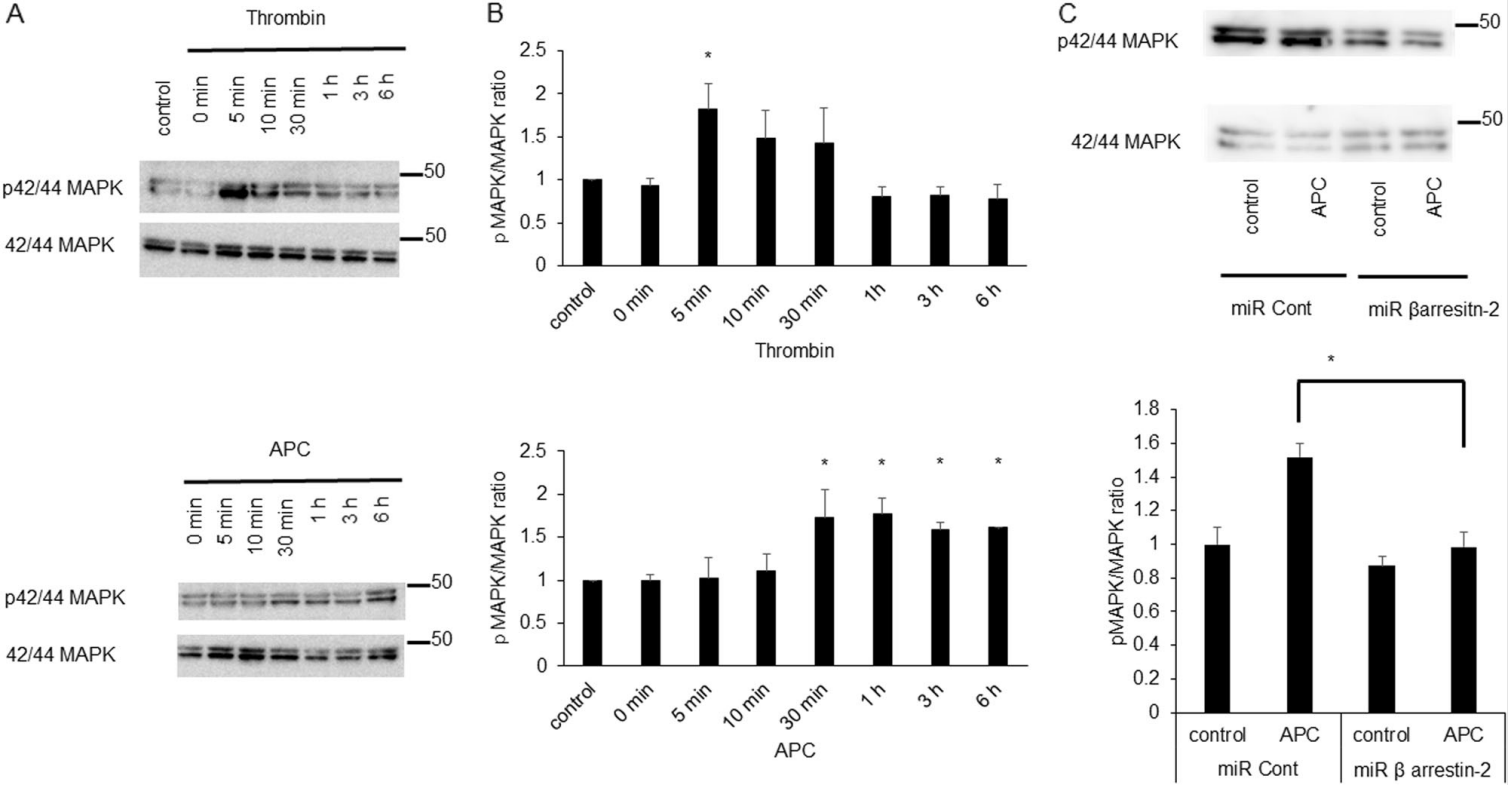
The temporal profiles of MAPK 42/44 phosphorylation by thrombin or APC with time. **a** Upper panel showed western blot analysis of phosphorylation of MAPK 42/44 under the treatment with thrombin 0.5 U/mL and lower panel showed under the treatment with APC 1.0 ng/mL. **b** Quantitative results of western blot analysis of the treatment with thrombin (upper panel) and APC (lower panel). Results are means ± SEM (*n* = 4). *p < 0.05 compared with the HUVECs under no treatment by Mann–Whitney *U*-tests. **c** Upper panel showed western blot analysis using β-arrestin-2 knockdown cells (miR βarrestin-2) or control cells (miR Cont) under no treatment or treatment with APC 1.0 ng/mL. Lower panel showed quantitative results of western blot analysis using β-arrestin-2 knockdown cells (miR β arrestin-2) (*n* = 4) compared with control cells (miR Cont) (*n* = 4) under treatment with APC. Results are means ± SEM (*n* = 4). Data were analyzed compared with multiple groups by Kruskal–Wallis tests. *p < 0.05 compared with control cells under treatment of APC

To elucidate the PAR-1-biased signaling, we evaluated the phosphorylation of MAPK 42/44, a downstream kinase of PAR-1^37^. Thrombin (0.5 U/mL) phosphorylated MAPK rapidly with a peak at 5 min, followed by a decline to basal levels after 1 h (Fig. 3a, b). In contrast, APC (1 ng/mL) significantly increased phosphorylated MAPK after 30 min, followed by a constant increase phosphorylation level until 6 h (Fig. 3a, b). The effect of APC was almost same under treatment with higher concentration of APC (3 μg/mL) (Supplementary Fig. 2). The increase of MAPK phosphorylation by APC was suppressed in β-arrestin-2 knockdown cells (Fig. 3c).

Under ischemic condition, both MAPK phosphorylation and expression of PDGF-β were upregulated. However, these were decreased in the β-arrestin-2 knockdown cells (Supplementary Fig. 3). These results suggested MAPK 42/44 was downstream of β-arrestin-2 signaling. Thus, the different dynamics of cleaved PAR-1 might explain the contradictory effects of thrombin and APC on PAR-1-biased signaling.

### GLase-PAR1 as an indicator of PAR-1 cleavage activity by thrombin and APC

PAR-1 is a tethered-ligand receptor and PAR-1 extracellular domain has two distinct PAR1-cleavage sites at arginine residues (R41 and R46) that are differentially recognized by thrombin and APC, resulting in contradictory responses^35,38^.

A previous study suggested that PAR-1 was cleaved at a slower rate by APC than by thrombin on endothelium^39^. The delayed and sustained activation of MAPK 42/44 by APC might be related to the slow cleavage of PAR-1 by APC. However, PAR-1 cleavage activity was not monitored in real time, and the cleavage rate by APC is unclear.

**Fig. 4:**
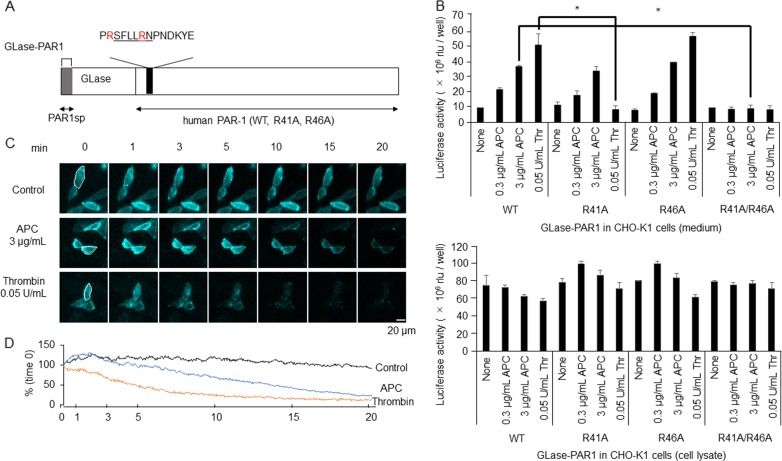
Quantitative bioluminescence analyses of PAR-1 cleavage activities by APC and thrombin in living cells using GLase-PAR1 as a reporter protein. **a** Schematic representation of a fusion protein of *Gaussia* luciferase and human PAR-1 (GLase-PAR1; also see Supplementary Fig. 4). The amino acid sequence of SFLLRN (underlined) is the tethered-ligand sequence. R41 and R46 residues in the human PAR-1 sequence are red-colored. When GLase-PAR1 expressed on the surface of living cells was cleaved by APC or thrombin, GLase was expected to be released from the cells; the luminescence activity of GLase in the culture medium and on the cell-surface were expected to increase and decrease, respectively. **b** Luminescence activities of the culture medium and cell lysate of CHO-K1 cells transiently expressing GLase-PAR1 and EPCR were determined by a luminometer. Cells transiently transfected with the expression vectors of GLase-PAR1 (R41/R46 wild-type or R41A-, R46A-, or R41A/R46A-double mutants) and EPCR were incubated for 30–60 min in HBSS containing 0.01% BSA with APC (0.3–3 µg/mL) or thrombin (0.05 U/mL). Luminescence activities in the culture medium and the cell lysate were determined by a Centro XS3 LB960 luminometer. Luminescence activity per well was expressed as the mean values ± S.D. (*n* = 8, *p < 0.05 compared with all multiple groups by Kruskal–Wallis tests). **c**, **d** Bioluminescence imaging analysis of GLase-PAR1 cleaved by APC and thrombin on the surface of living CHO-K1 cells. Based on the luminescence reaction of GLase and a luciferin (coelenterazine; 1 µg/mL), proteins on the cell surface were visualized at 2 s/frame using a microscopic system equipped with a water-cooled EM-CCD camera. Under these imaging conditions, the time-dependent decay in GLase activity was very slow and continuously visualized for 20 min after the start of recording. CHO-K1 cells transiently transfected for 48 h with the expression vectors of GLase-PAR1 and EPCR were washed with HBSS and pre-incubated for 10 min in the presence of coelenterazine. After incubation with APC (3 µg/mL) or thrombin (0.05 U/mL) for 10 s, luminescence signals of GLase-PAR1 were recorded for 20 min with an exposure time of 2 s. The luminescence images of GLase-PAR1 on the cell-surface (colored cyan) at 0, 1, 3, 5, 10, 15, and 20 min were shown. Scale bar was 20 µm. **d** Luminescence intensities of video image in a cell area (white circle in **c**) were calculated. Error bars represent ±SD (*n* = 8–10)

To analyze the cleavage rate of PAR-1 on the surface of living cells, we applied real-time bioluminescence imaging to visualize secreted and cell surface-bound proteins^14–16,29,30,40,41^. GLase is a suitable reporter protein to analyze proteins on the outside of the cell surface, because it exhibited high luminescence activity when expressed in the ER-Golgi pathway of mammalian cells using codon-optimized GLase genes^27,28,42–44^. We used a fusion protein of GLase and human PAR-1 (GLase-PAR1) (Fig. 4a and Supplementary Fig. 4).

To evaluate whether GLase-PAR1 is a suitable reporter protein for analysis of PAR-1 cleavage activity, GLase-PAR1 was transiently expressed in HEK293, CHO-K1, and endothelial bEnd.3 cells incubated with APC or thrombin, and the luminescence activity of GLase released into the culture medium was determined using a luminometer (Fig. 4b, Supplementary Figs. 5 and 6).

In HEK293 cells, GLase-PAR1 was transiently expressed with or without EPCR and incubated with APC or thrombin (Supplementary Fig. 5A). The luminescence activity of GLase released into the culture medium was increased by thrombin, regardless of the co-expression of EPCR. The luminescence activity of GLase in the culture medium increased 3.5-fold by APC in the cells that expressed GLase-PAR-1 with EPCR, whereas the increse was only 1.5-fold without EPCR. In the medium of HEK293 cells co-expressing GLase-PAR1 and EPCR, the GLase activity was increased by APC in a dose-dependent manner (Supplementary Fig. 5B). In bEnd.3 cells transiently expressing both GLase-PAR1 and EPCR, the luminescence activity of released GLase increased substantially (Supplementary Fig. 6). These results indicated that GLase-PAR1 was cleaved by APC and thrombin when co-expressed with EPCR.

### Cleavage of GLase-PAR1 by thrombin and APC at specific sites

CHO-K1 cells expressed low level of native GPCR and channels and overexpression of GPCR in CHO-KI cells allowed us to clarify the dynamics of GPCR. Then, GLase-PAR-1 variants with R41A, R46A, and R41A/R46A-double mutations in the PAR-1 sequence were transiently co-expressed with EPCR in CHO-K1 cells (Fig. 4a, b and Supplementary Fig. 4). In cells expressing wild-type GLase-PAR1, luminescence activity of GLase released into medium was increased by thrombin and APC (Fig. 4b, upper panel). Consistent with the GLase release, luminescence activity in cell lysate of wild-type GLase-PAR1 decreased after APC or thrombin (Fig. 4b, lower panel). In cells expressing R41A or R41A/R46A double mutants of GLase-PAR1, GLase release induced by thrombin was completely suppressed, but not R46A mutant cells (Fig. 4b). In contrast, the release of GLase induced by APC was completely suppressed only in R41A/R46A-double mutant cells (Fig. 4b). Thus, GLase-PAR1 was a suitable reporter protein for the detection of PAR-1 cleavage activity by bioluminescence.

### Slower cleavage of PAR-1 by APC than thrombin in real time by bioluminescence imaging

**Fig. 5:**
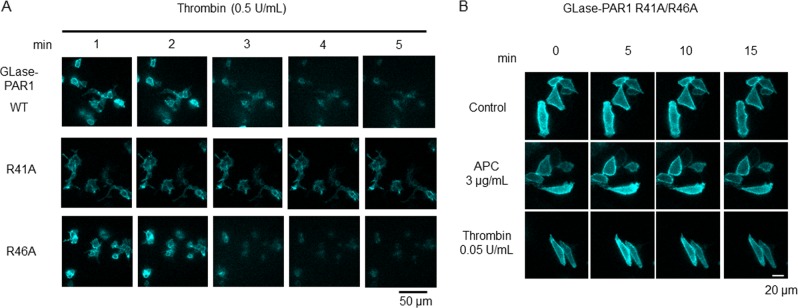
Quantitative bioluminescence analyses of PAR-1 cleavage activities by APC and thrombin in living cells using GLase-PAR1 mutant cells. **a** Bioluminescence imaging analysis of GLase-PAR1 cleaved by thrombin on the surface of living HEK293 cells. HEK293 cells were transiently transfected for 24 h with the expression vectors of GLase-PAR1 wild type, R41A-, or R46A- mutants. After incubation with thrombin (0.5 U/mL) for 10 s, luminescence signals of GLase-PAR1 were recorded for 5 min with an exposure time of 2 s. The luminescence images of GLase-PAR1 on the cell-surface (colored cyan) at 1, 2, 3, 4, and 5 min were shown. Scale bar was 50 µm. **b** Bioluminescence imaging analysis of GLase-PAR1 cleaved by thrombin or APC on the surface of living CHO-K1 cells. Cells were transiently transfected for 24 h with the expression vectors of both the GLase-PAR1 R41A/R46A double mutant and EPCR. After incubation with thrombin (0.05 U/mL) or APC (3 μg/mL) or control for 10 s, luminescence signals of GLase-PAR1 were recorded for 15 min with an exposure time of 2 s. The luminescence images of GLase-PAR1 on the cell-surface (colored cyan) at 0, 5, 10, and 15 min were shown. Scale bar was 20 µm

To analyze the cleavage rate of PAR-1, GLase-PAR1 on the surface of living cells was visualized by quantitative bioluminescence video imaging^15,16,29,30,41^. In cells treated with APC, the luminescence signals gradually disappeared over 20 min (Fig. 4c). In contrast, the signals disappeared rapidly in a period of 5–10 min after thrombin treatment. The decrease in luminescence intensity induced by APC or thrombin in the video images corresponded to the cleavage activity of GLase-PAR1 by these proteases. Luminescence intensity in the cell area on the video images was used as an indicator to quantity the cleavage rate of GLase-PAR1. The decrease in luminescence intensity by APC was slower than that by thrombin (Fig. 4d). The decrease in luminescence intensity induced by thrombin was detected in transiently expressing wild-type PAR-1 and R46A mutant cells without EPCR, but not detected in R41A mutants (Fig. 5a). In cells expressing the R41A/R46A double mutant of PAR-1 co-expressing with EPCR, we found the sustained luminescence signals of GLase-PAR1 on the cell surface (Fig. 5b), consistent with no increase in the luminescence activity of GLase released into the medium (Fig. 4b). These results clearly showed the specific cleavage of PAR-1 at R41 and R41/R46 by thrombin and APC, respectively.

### Downstream target molecules for angiogenesis mediated by β-arrestin-2

**Fig. 6:**
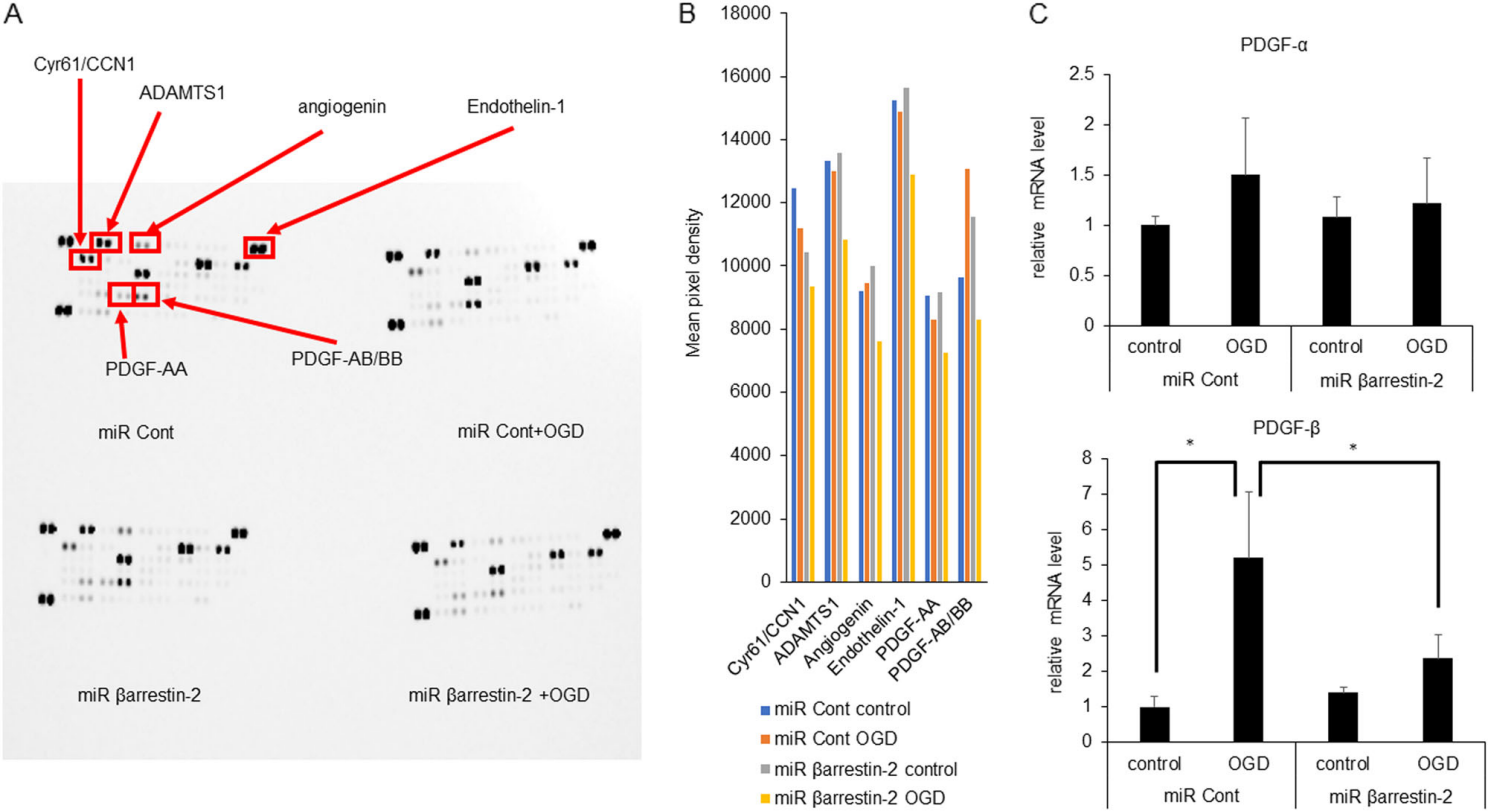
Essential molecules promote angiogenesis mediated by β-arrestin-2-dependent pathway with or without ischemia. **a** Protein array related to angiogenesis was examined using β-arrestin-2 knockdown cells (miR βarrestin-2) under ischemic condition (1% O_2_ 3 h) compared with control cells (miR Cont). After 18 h from OGD treatment, representative array membranes showed. Six of total 31 angiogenesis-related proteins with significant change were marked with square boxes and labeled. **b** Densitometric analysis revealed low level of Cyr61/CCN, AMDMTS1, Angiogenin, Endothelin-1, PDGF-AA and PDGF-AB/BB in β-arrestin-2 knockdown cells (miR β arrestin-2) compared with respective controls (miR Cont). **c** Results of quantitative RT-PCR of PDGF-α and PDGF-β at β-arrestin-2 knockdown cells (miR β arrestin-2) were showed compare with control HUVECs (miR Cont). Results are means ± SEM (*n* = 4). Data were analyzed compared with multiple groups by Kruskal–Wallis tests. *p < 0.05

We next identified proteins that were essential for the β-arrestin-2-dependent angiogenesis under ischemic condition, using a mouse angiogenesis array. The levels of 6 of 31 angiogenesis-related proteins were affected by the knockdown of β-arrestin-2 with or without ischemia (Fig. 6a, b). We evaluated these six molecules, Cyr61/CCN1, ADMTS1, Angiogenin, Endothelin-1, PDGF-AA and PDGF-AB at the mRNA levels by quantitative RT-PCR analysis (qPCR). PDGF-β was upregulated by OGD compared to that of untreated cells (Fig. 6c) and this upregulation was lower in β-arrestin-2 knockdown cells compared with control cells. PDGF-α level did not differ significantly under ischemic conditions (Fig. 6c). These results showed that PDGF-β was upregulated via a β-arrestin-2-dependent pathway under ischemic condition.

### Relationship between β-arrestin-2 and PDGF-β secretion as well as gene expression

To examine whether β-arrestin-2 is required for the secretion of PDGF-β, a fusion protein of PDGF-β and GLase (PDGF-β-GLase) was used as a reporter protein (Supplementary Figs. 7 and 8A). The miRNA targeting β-arrestin-2 suppressed the luminescence activity of PDGF-β-GLase secreted into the medium in APC- and thrombin-treated cells, but the fusion protein secretion was not significantly increased by treatment with these proteases (Supplementary Fig. 8B). Additionary, the miR-β arrestin-2 significantly inhibited PDGF-β secretion by APC than by thrombin. In contrast, miR-β arrestin-2 did not inhibited the luminescence activity of GLase with its signal peptide sequence (GLsp-GLase: Supplementary Fig. 8C) secreted into the medium (Supplementary Fig. 8D). PDGF-β was significantly upregulated under treatment with APC by quantitative RT-PCR analysis (Supplementary Fig. 9). MEK inhibitors, U0126 and PD98059, were inhibited this APC-induced PDGF-β upregulation (Supplementary Fig. 9). These results suggest that the activation of PAR-1 in complex with EPCR by APC and the resulting β-arrestin-2-MAPK 42/44 signaling were essential for PDGF-β gene expression as well as its secretion.

### Exacerbation of ischemic stroke at HFD mice due to the disruption of BBB integrity

**Fig. 7:**
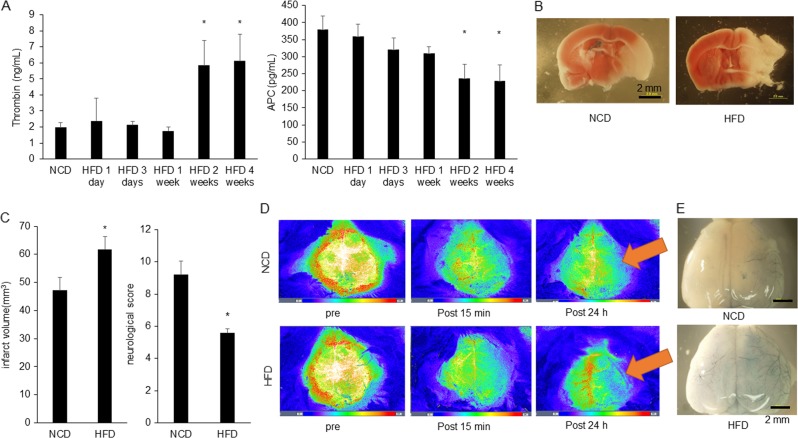
HFD feeding mice show larger infarct volume and more severe neurological score than NCD mice. **a** ELISA analysis of thrombin and APC at HFD mice or NCD mice were shown. Left panel showed quantitative results of thrombin concentration (*n* = 5–7). Results are means ± SEM. *p < 0.05 compared with NCD mice by Mann–Whitney *U*-tests. Right panel showed quantitative results of APC concentration (*n* = 8–10). Results are means ± SEM. *p < 0.05 compared with NCD mice by Mann–Whitney *U*-tests. **b** Representative TTC staining after MCAO of HFD feeding mice and NCD mice. Scale bar was 2 mm. **c** Left panel showed of infarct volume of NCD mice and HFD mice (NCD, 47.2 ± 4.6 mm^3^, *n* = 5; HFD, 61.8 ± 4.5 mm^3^, *n* = 5; *p* < 0.05 by Mann–Whitney *U*-tests). Results are means ± SEM. Right panel showed neurological score of NCD mice and HFD mice (NCD, 9.2 ± 0.4, *n* = 5; HFD, 5.6 ± 0.1, *n* = 5; *p* < 0.05 by Mann–Whitney *U*-tests). Results are means ± SEM. **d** Representative blood flow images of transient MCAO at pre-operation, after 15 min and after 24 h from MCAO using Omagazone. Upper panel showed blood flow of NCD feeding and lower panel showed blood flow of HFD feeding mice. Scale bar was 2 mm. **e** Representative image of transient MCAO brain after Evans Blue injection intravenously from tail vein. Scale bar was 2 mm

HFD-fed rats showed elevated endogenous thrombin potential^22^, therefore we examined endogenous thrombin and APC in high fat diet (HFD) fed mice and normal chow diet (NCD) fed mice using ELISA. Two weeks after HFD feeding, HFD mice showed more thrombin and less APC compared with NCD mice (Fig. 7a). We used HFD model in vivo as it is a seemingly suitable model to reproduce PAR-1-biased signaling obtained from in vitro studies. Then, we performed transient MCAO. HFD mice showed a larger infarct volume (Fig. 7b, c) and more severe neurological findings (Fig. 7c) than those of NCD mice. We performed a real-time analysis of cerebral blood flow (CBF) using Omegazone during transient MCAO until 15 min after the procedure, and CBF levels were approximately the same in both groups. However, at 1 day after ischemia, CBF in HFD mice was lower than NCD groups (Fig. 7d). We then assessed BBB dysfunction using a permeability assay with Evans Blue at 24 h after MCAO. HFD mice showed larger leakage volume of Evans Blue into the cerebral parenchyma than NCD groups (Fig. 7e and Supplementary Fig. 10). These results suggested that aggravating ischemic stroke in HFD mice were caused by damage to the BBB integrity.

### Lower levels of β-arrestin-2 and PDGF-β in HFD mice than in NCD mice

**Fig. 8:**
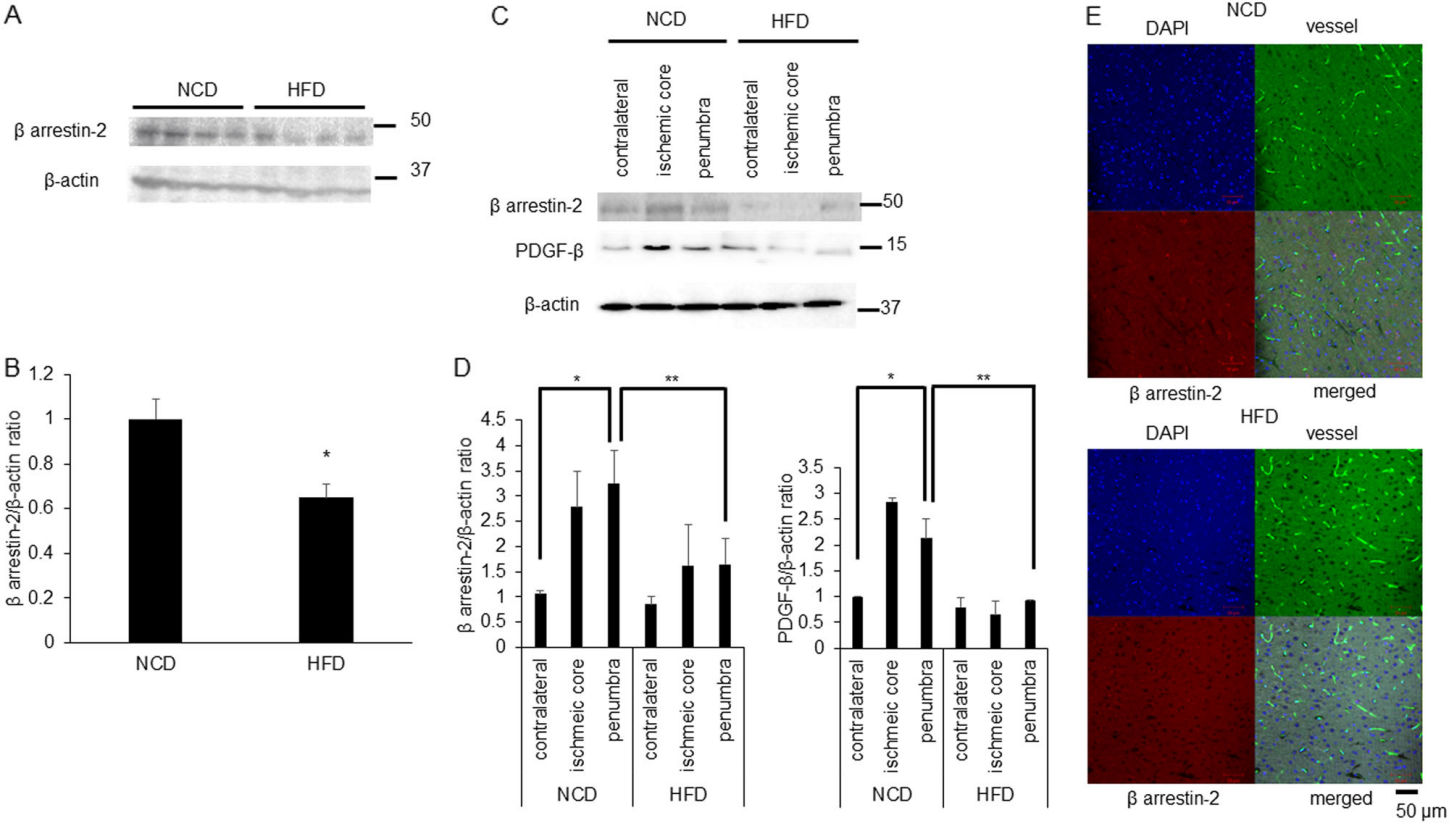
β arrestin-2 and PDGF-β are less expression level at penumbra lesion in HFD mice compared with normal control diet (NCD) mice. **a** This panel showed western blot analysis of β-arrestin-2 and β-actin at HFD mice compared with NCD mice under no treatment. **b** Quantitative results. β-arrestin-2/β-actin ratio compared with NCD mice were shown (NCD mice 1.0 ± 0.09; HFD mice 0.65 ± 0.06, *n* = 4) and results are means ± SEM. *p < 0.05 compared with NCD mice by Mann–Whitney *U*-tests. **c** This panel showed western blot analysis of β-arrestin-2, PDGF-β and β-actin at contralateral lesion, ischemic core lesion and penumbra lesion after 48 h from transient MCAO. **d** Quantitative results. β-arrestin-2/β-actin ratio and PDGF-β/β-actin ratio compared with contralateral lesion of NCD mice after 48 h MCAO of NCD and HFD feeding mice were shown. Results are means ± SEM (*n* = 4). Data were analyzed compared with all multiple groups by Kruskal–Wallis tests. *p < 0.05. **f** Representative image of immunostaining in the brains at penumbra lesion of MCAO mice. The nucleus appears blue due to DNA staining with DAPI and vessel appears green with fluorescence and β-arrestin-2 appears red. Scale bar was 50 µm

HFD mice showed lower levels of β-arrestin-2 compared with those in NCD mice (Fig. 8a, b). Palmitic acid treatment has been used as a HFD model in vitro^45^. We found that a high concentration of palmitic acid (100 µM) was associated with reduced β-arrestin-2 (Supplementary Fig. 11). We observed significant increases in the expression levels of β-arrestin-2 and PDGF-β in penumbra lesions of NCD mice 48 h after MCAO than in ipsilateral lesions; these protein levels were significantly lower at penumbra lesions of HFD mice compared with NCD mice (Fig. 8c, d). The residual perfusion of the microcirculation after MCA occlusion was assessed using dichlorotriazinyl amino fluorescein (DTAF)^46^. In the penumbra lesion, capillary fluorescence after MCAO indicated that the expression of β-arrestin-2 was lower in HFD mice than in NCD mice (Fig. 8e). These results suggested that β-arrestin-2 plays an important role in endothelial functions mediated by PDGF-β after stroke in HFD-induced obesity (Supplementary Fig. 12).

## Discussion

Our results revealed that β-arrestin-2 was a key molecule in PAR-1-biased signaling and PAR-1-β-arrestin-2-MAPK 42/44 signaling had a protective effect on endothelial function via PDGF-β. Furthermore, we demonstrated for the first time that the cleavage rate of PAR-1 by APC was slower than by thrombin, using the GLase-PAR1 reporter protein, as determined by quantitative bioluminescence imaging.

Recently, the discovery of molecules with the capacity to activate distinct pathways after interacting with the same GPCR suggested the importance of functional selectivity and biased agonism. Agonists capable of preferentially triggering β-arrestin-dependent signaling pathways were referred to as β-arrestin-biased agonists, and those capable of preferentially triggering G-protein-dependent signaling pathways were referred to G-protein-biased agonists^47–51^.

When thrombin binds to endothelial cell surface thrombomodulin, this protease-cofactor complex no longer functions in the procoagulant pathway, but instead initiates the anticoagulant pathway by APC in complex with EPCR^52–56^. Our findings suggested that APC in complex with EPCR as previous study using a fusion protein of secreted alkaline phosphatase and PAR-1^39^ and, consequently, β-arrestin-2-MAPK-PDGF-β signaling in PAR-1 biased signaling are essential for endothelial protection. The β-arrestin-1 has important preventive roles under ischemic conditions^57^; however, in our study, β-arrestin-1 levels were not affected by stroke or HFD-induced obesity. In neurons, β-arrestin-1 was upregulated under ischemic conditions^57^, on the other hands, we used HFD model focusing endothelial cells in our study. These reasons might lead to the different results. Recently, β-arrestin-2 was reported to be playing a dominant role after ischemic stroke^58^, consistent with our reports.

Furthermore, we observed the difference in the time-dependent cleavage of PAR-1 by APC and thrombin. The slower cleavage rate by APC was paralleled by the slow and sustained activation of MAPK 42/44 signaling by APC compaered with thrombin, explaining the difference in the effects of thrombin and APC on PAR-1-biased signaling. The occupancy of EPCR by protein C switches the specificity of PAR-1-dependent transduction by thrombin from a permeability-enhancing to a barrier-protective response^59^. Consistent with previous reports, our results showed that thrombin cleaved PAR-1 within 5 min, and APC cleaved PAR-1 slowly over about 30 min^60^. Parathyroid hormone, β2 adrenergic receptor and angiotensin type 1 A receptor showed an early and a later sustained ERK activation and G proteins were related to an early, but a later activation was related to β-arrestin^61–64^. The sustained enhancement of phosphorylated MAPK lead to different responses to the transient activation of ERK^65^. This sustained activation of MAPK 42/44 induced by APC might explain the different effects on PAR-1-biased signaling. Akt signaling was also related to the effect of APC^66,67^. ERK1/2 and Akt were related to angiogenesis in a PAR-1-dependent manner^68^.

We found PDGF-β was related to the β-arrestin-2-dependent pathway. The expression of PDGF-β increased rapidly after ischemic stroke, but the expression of PDGF receptor β increased slowly and peaked at 3–5 days after ischemic stroke. These results showed that PDGF-β was a key molecule for BBB breakdown^69^.

We detected high thrombin and low APC 2 weeks after HFD feeding, reproducing PAR-1-biased signaling. Fatty acids cross the blood–brain barrier mainly by simple diffusion. We found that saturated PA decreased β-arrestin-2 expression. HFD mice or saturated fatty acids showed reduced expression levels of β-arrestin-2 in capillaries of the penumbra lesions, leading to aggravate ischemic injury.

This is the first demonstration of the visualization of proteins cleaved by proteases on the surface of living cells in real-time. In addition, this is also the first report to visualize GPCR by bioluminescence in living cells. Our bioluminescence imaging method using GLase-PAR1 is useful to investigate the relation between the cleavage rate of PAR-1 by proteases and the biased signaling. We also demonstrated that PAR-1 cleavage activity could be determined using a luminometer to measure the luminescence activity of GLase released into the culture medium. The GLase-PAR1 reporter protein might be useful for studies of diseases related to PAR-1.

Our study had some limitations. First, we cannot completely separate the β-arrestin-2-dependent pathway from G-proteins-dependent pathway and thrombin only partially activates β-arrestin-2-dependent pathway. Second, we cannot be ruled out the lack of correlation of G-protein pathways under the effect of APC. Third, we used HFD mice, which showed low expression level of β-arrestin-2. However, in addition to β-arrestin-2-indepedent signaling, HFD mice are characterized by systemic inflammation, changes in leptin levels, etc.

This study demonstrates that β-arrestin-2 in PAR-1-biased signaling plays a pivotal role in stroke or HFD-induced obesity by the enhancement of endothelial function and integrity via PDGF-β. Furthermore, this is the first study to visualize proteins cleavage by multiple proteases on the surface of living cells in real-time. Factor Xa also cleaved PAR-1^70-72^. Therefore, various direct oral anticoagulant factor Xa inhibitors may have differentially distinct effects on PAR-1-biased signaling. Further studies may lead to novel drug discovery for prevention or adjuvant pharmacological therapy together with endovascular therapy for ischemic stroke.

## Supplementary information

Supplementary Figure.1

Supplementary Figure.2

Supplementary Figure.3

Supplementary Figure.4

Supplementary Figure.5

Supplementary Figure.6

Supplementary Figure.7

Supplementary Figure.8

Supplementary Figure.9

Supplementary Figure.10

Supplementary Figure.11

Supplementary Figure.12

supplemental figure legends
