# Supplementary figures and images for: β-arrestin-2 in PAR-1-biased signaling has a crucial role in endothelial function via PDGF-β in stroke
Source: Cell Death Dis. 2019 Feb 4;10(2):100. doi: 10.1038/s41419-019-1375-x (PMC6361911; doi:10.1038/s41419-019-1375-x)

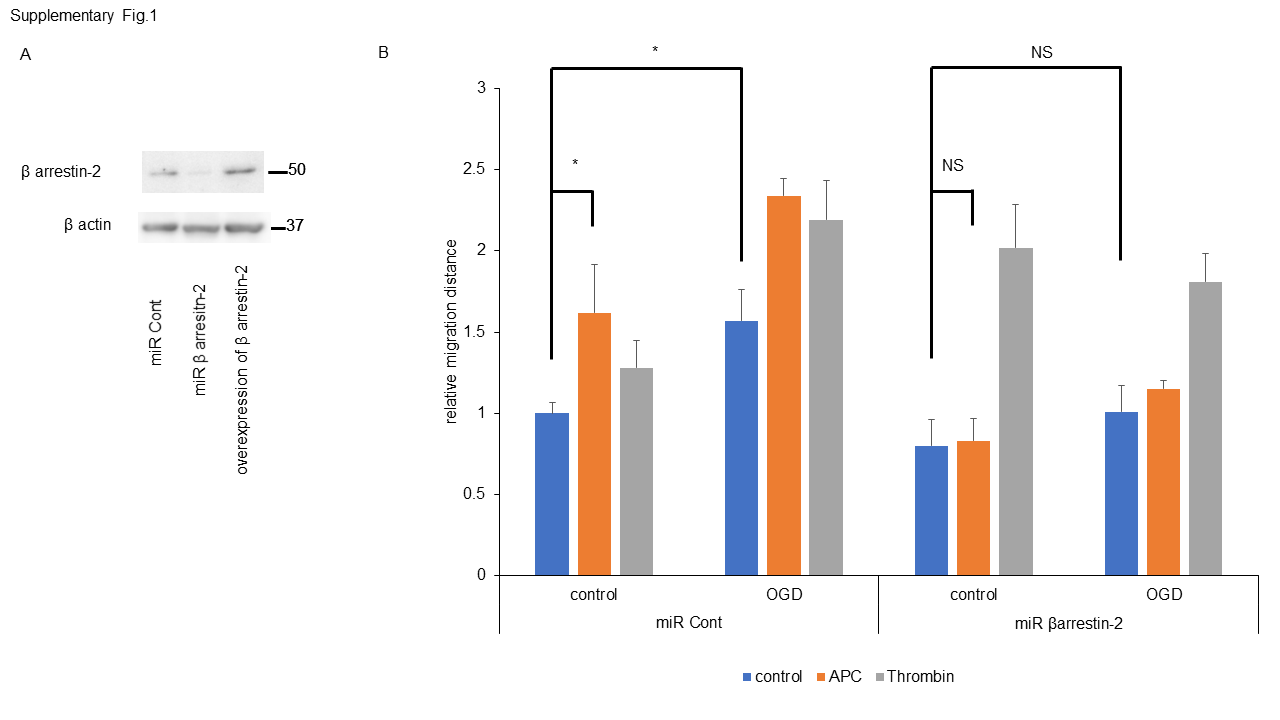

Supplement: Supplementary file 1 — Supplementary Figure.1 [file 41419_2019_1375_MOESM1_ESM.tif]

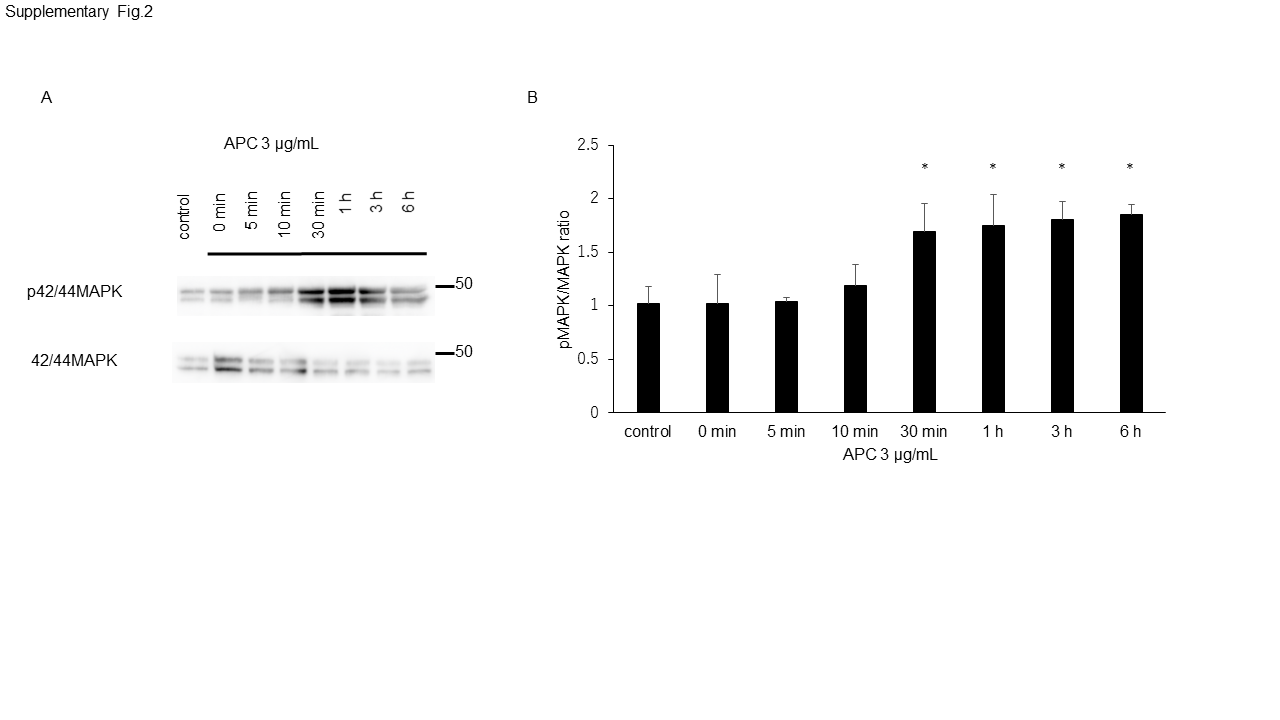

Supplement: Supplementary file 2 — Supplementary Figure.2 [file 41419_2019_1375_MOESM2_ESM.tif]

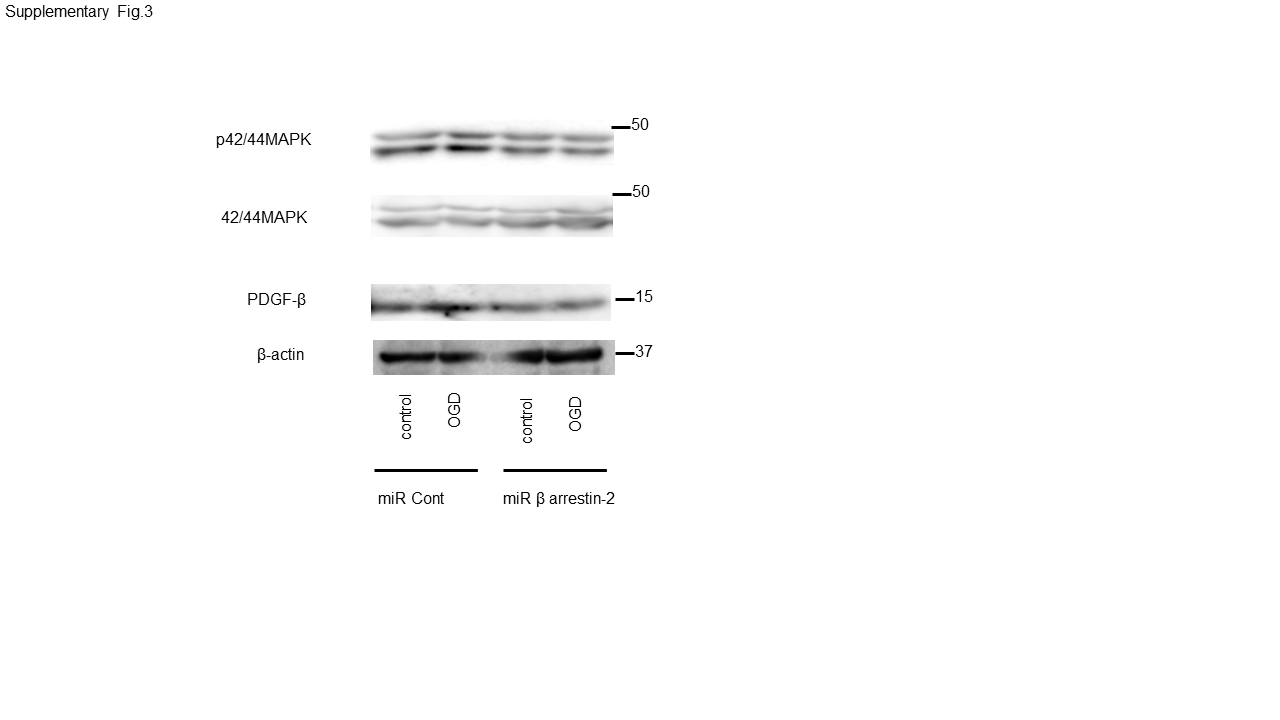

Supplement: Supplementary file 3 — Supplementary Figure.3 [file 41419_2019_1375_MOESM3_ESM.tif]

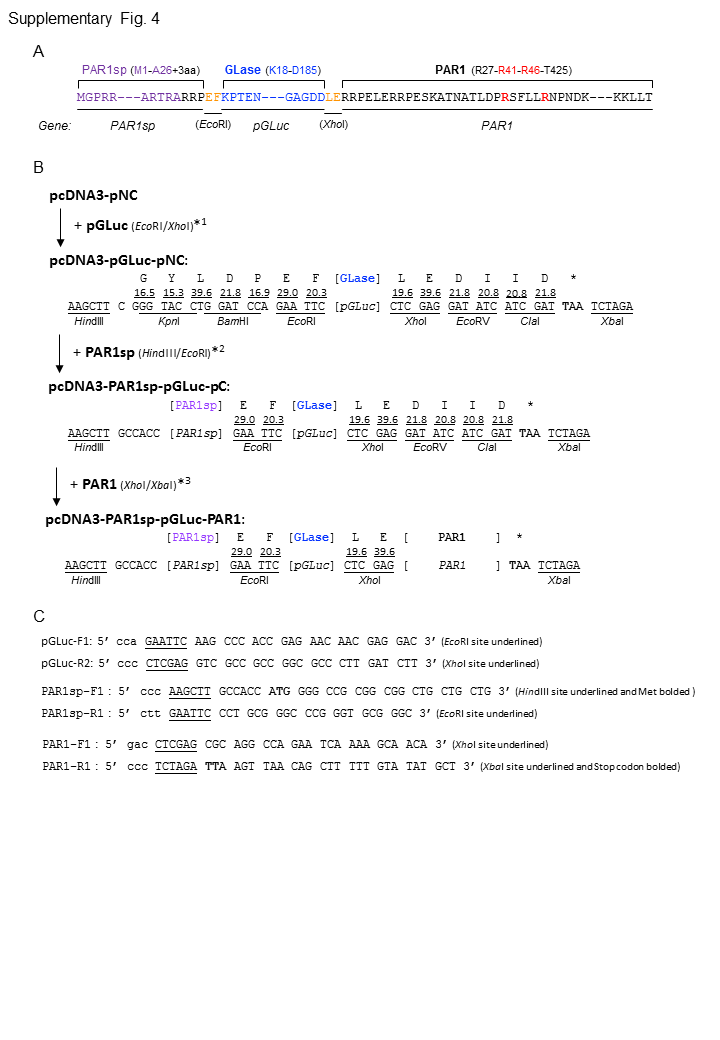

Supplement: Supplementary file 4 — Supplementary Figure.4 [file 41419_2019_1375_MOESM4_ESM.tif]

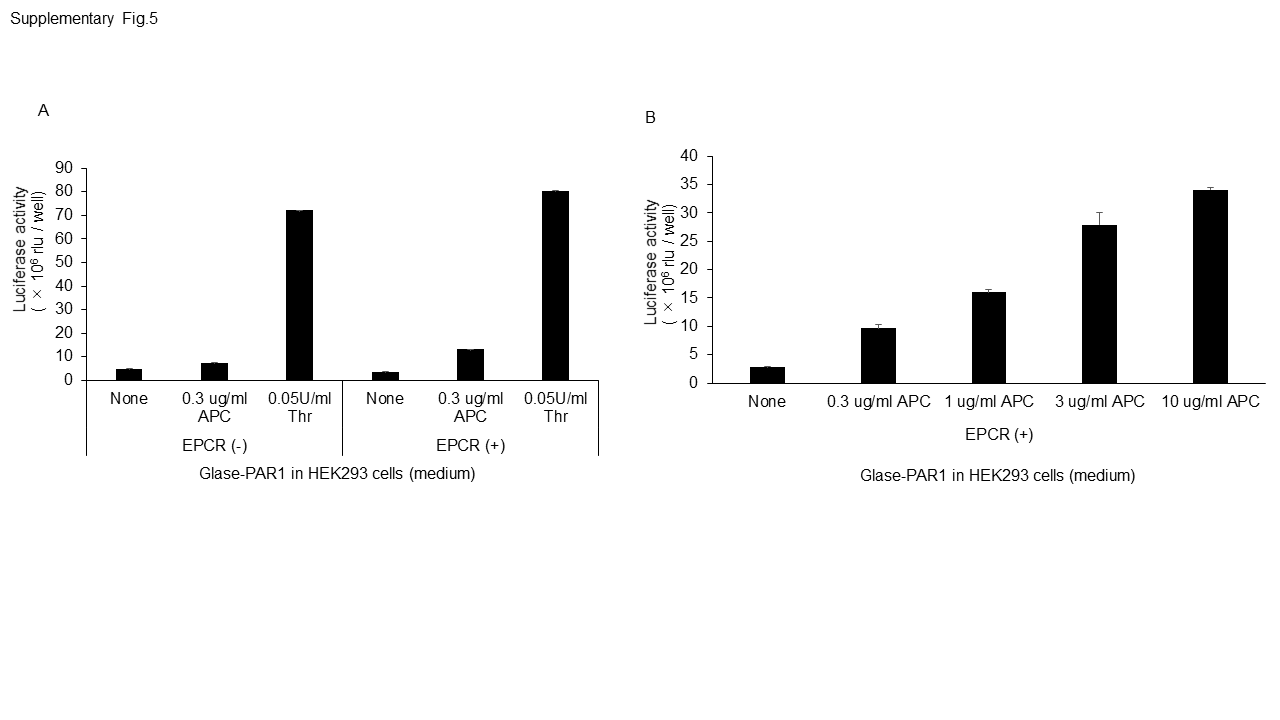

Supplement: Supplementary file 5 — Supplementary Figure.5 [file 41419_2019_1375_MOESM5_ESM.tif]

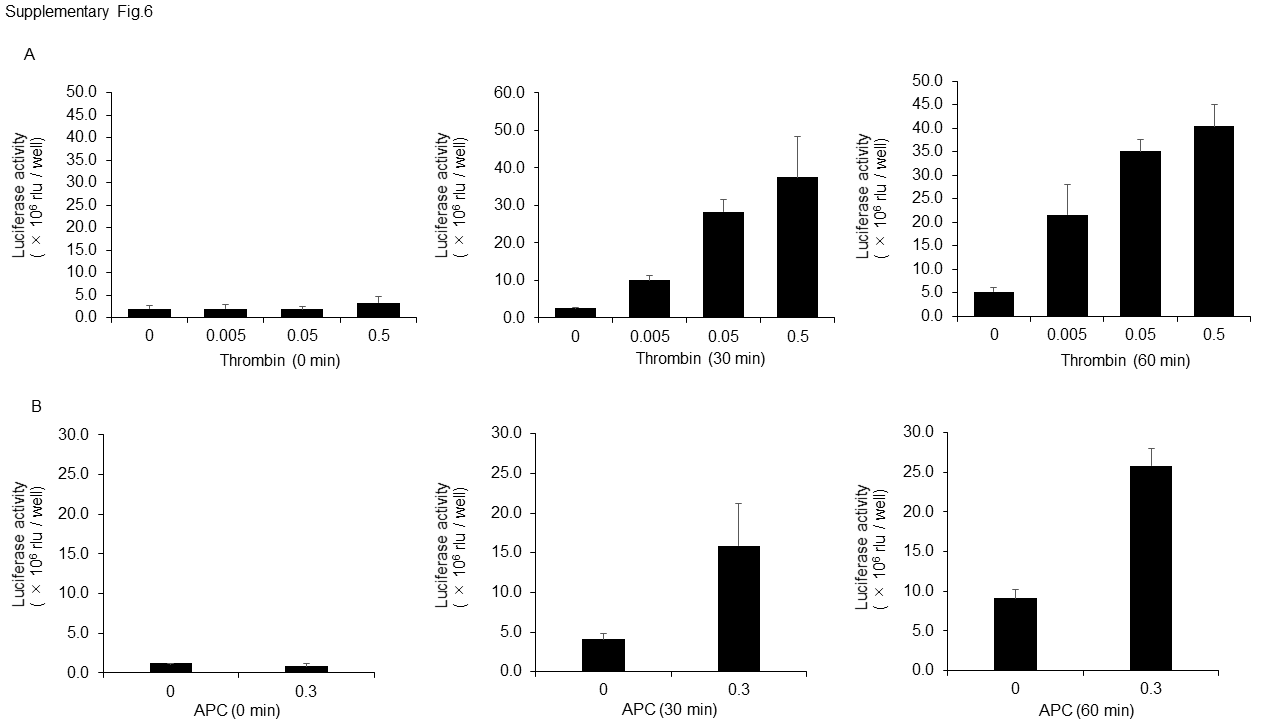

Supplement: Supplementary file 6 — Supplementary Figure.6 [file 41419_2019_1375_MOESM6_ESM.tif]

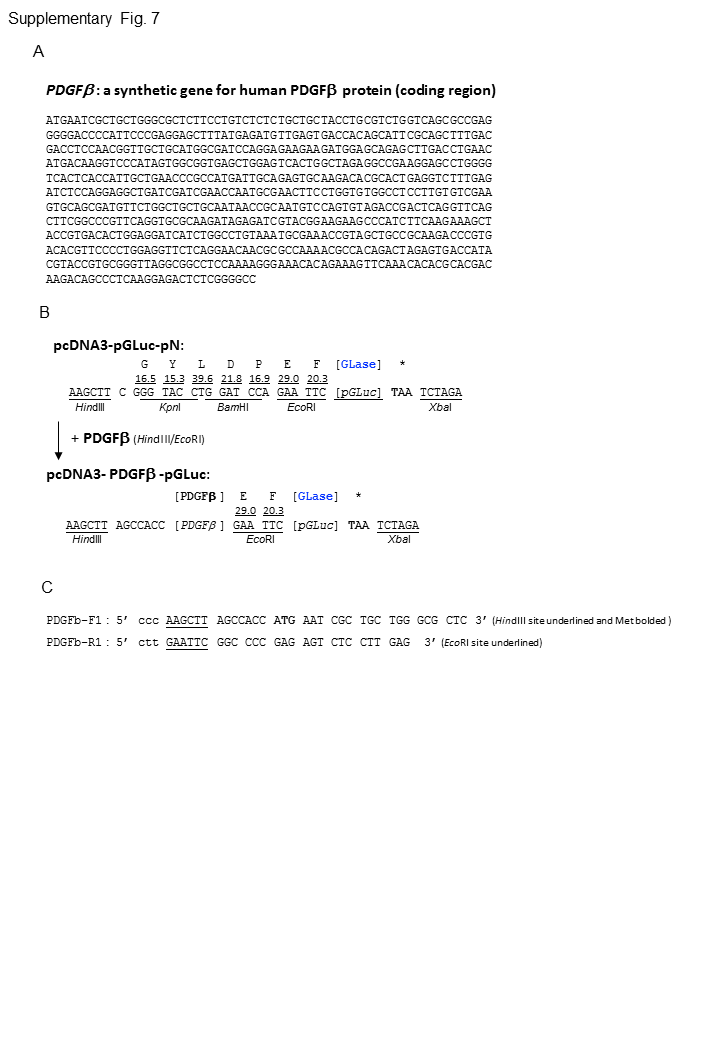

Supplement: Supplementary file 7 — Supplementary Figure.7 [file 41419_2019_1375_MOESM7_ESM.tif]

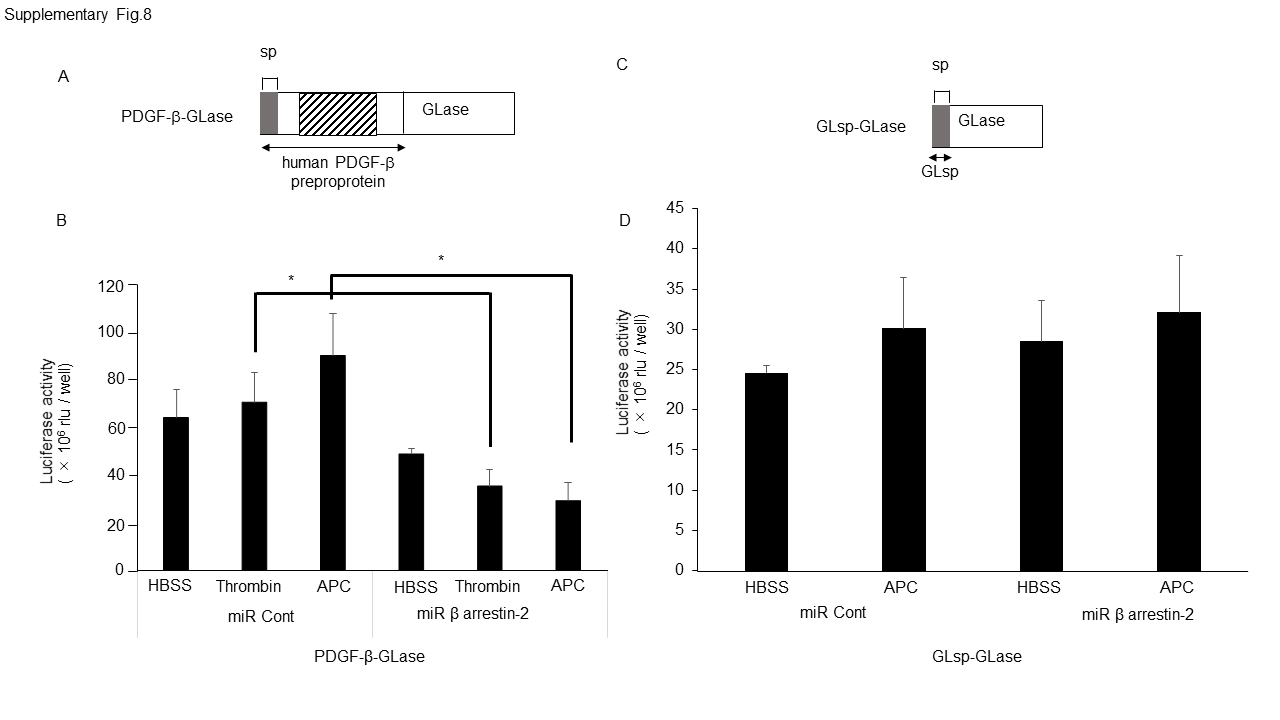

Supplement: Supplementary file 8 — Supplementary Figure.8 [file 41419_2019_1375_MOESM8_ESM.tif]

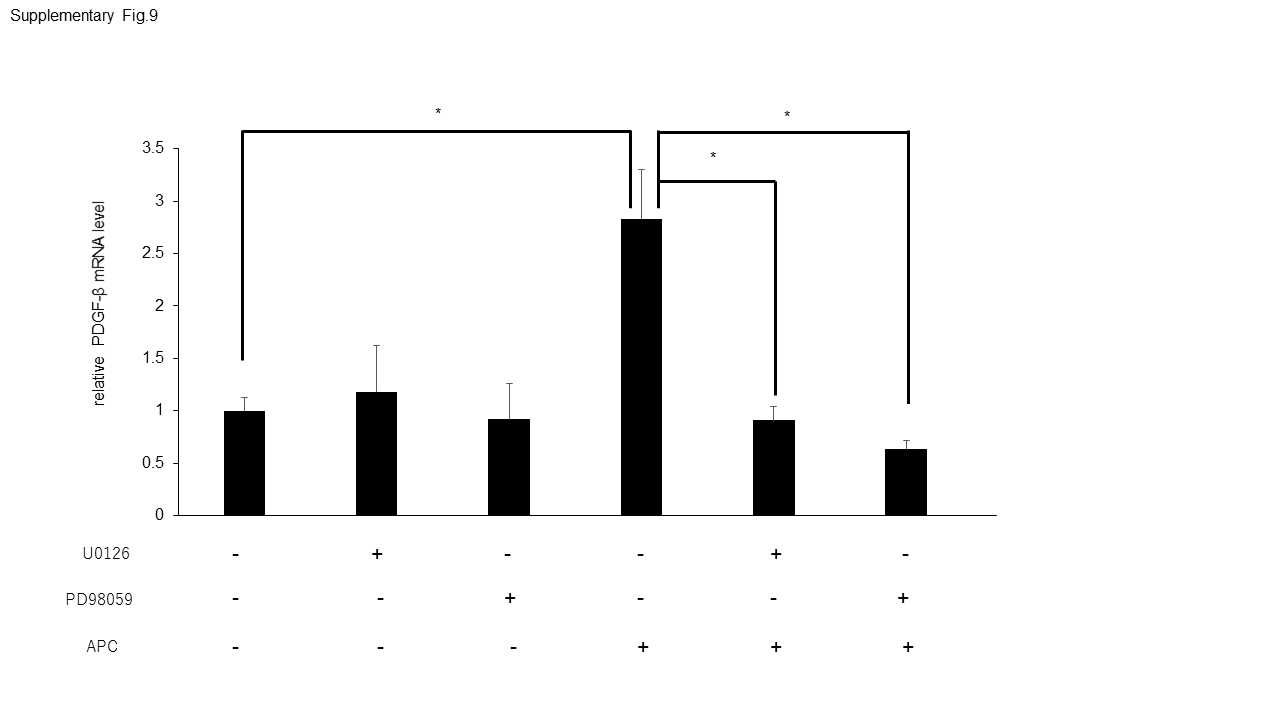

Supplement: Supplementary file 9 — Supplementary Figure.9 [file 41419_2019_1375_MOESM9_ESM.tif]

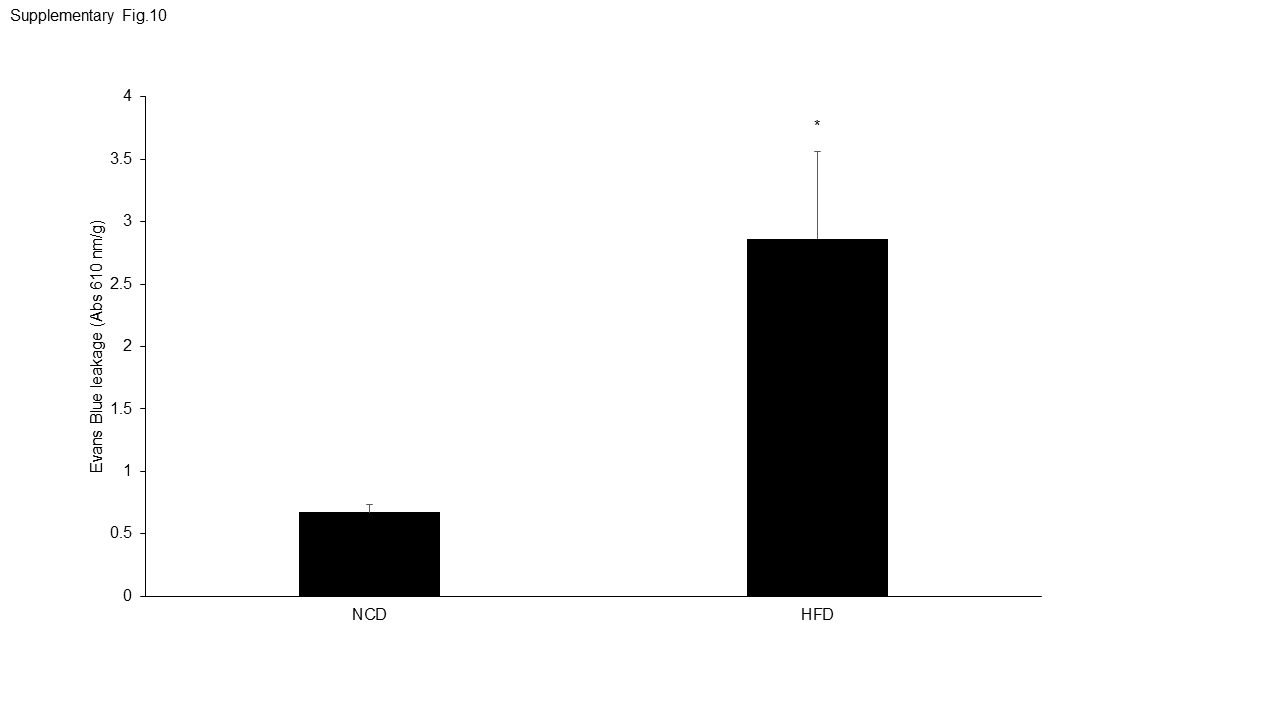

Supplement: Supplementary file 10 — Supplementary Figure.10 [file 41419_2019_1375_MOESM10_ESM.tif]

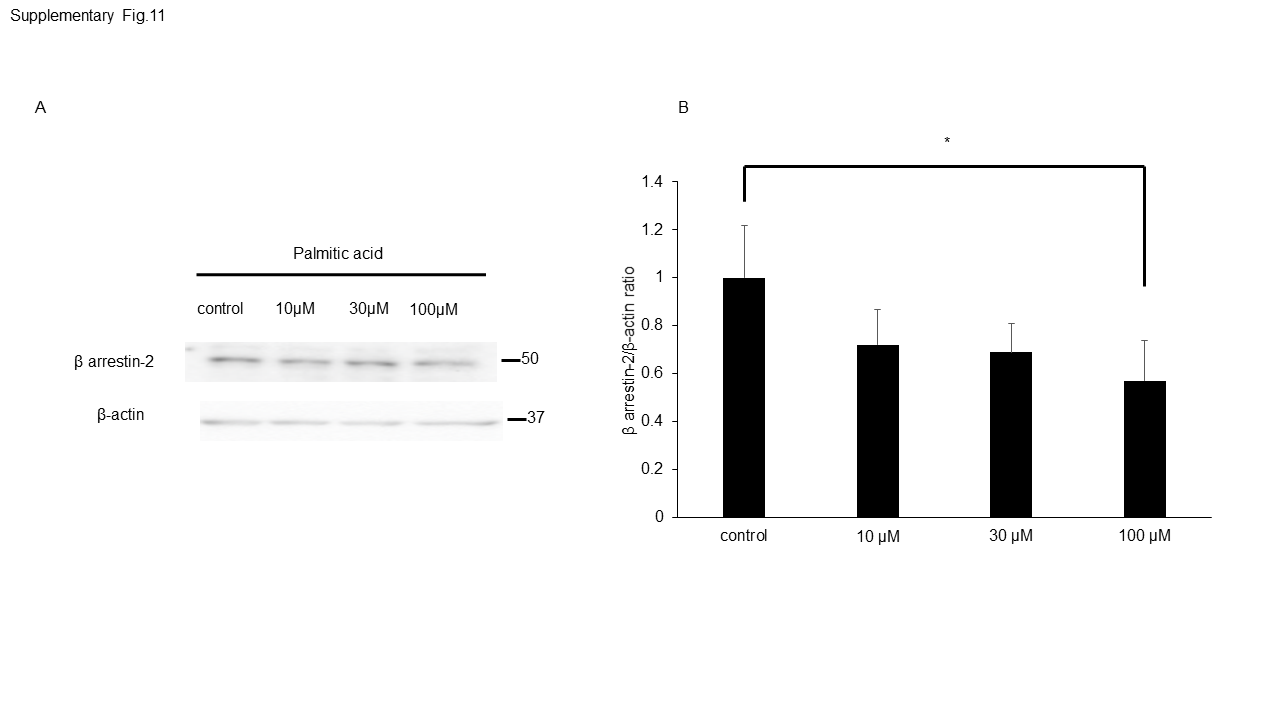

Supplement: Supplementary file 11 — Supplementary Figure.11 [file 41419_2019_1375_MOESM11_ESM.tif]

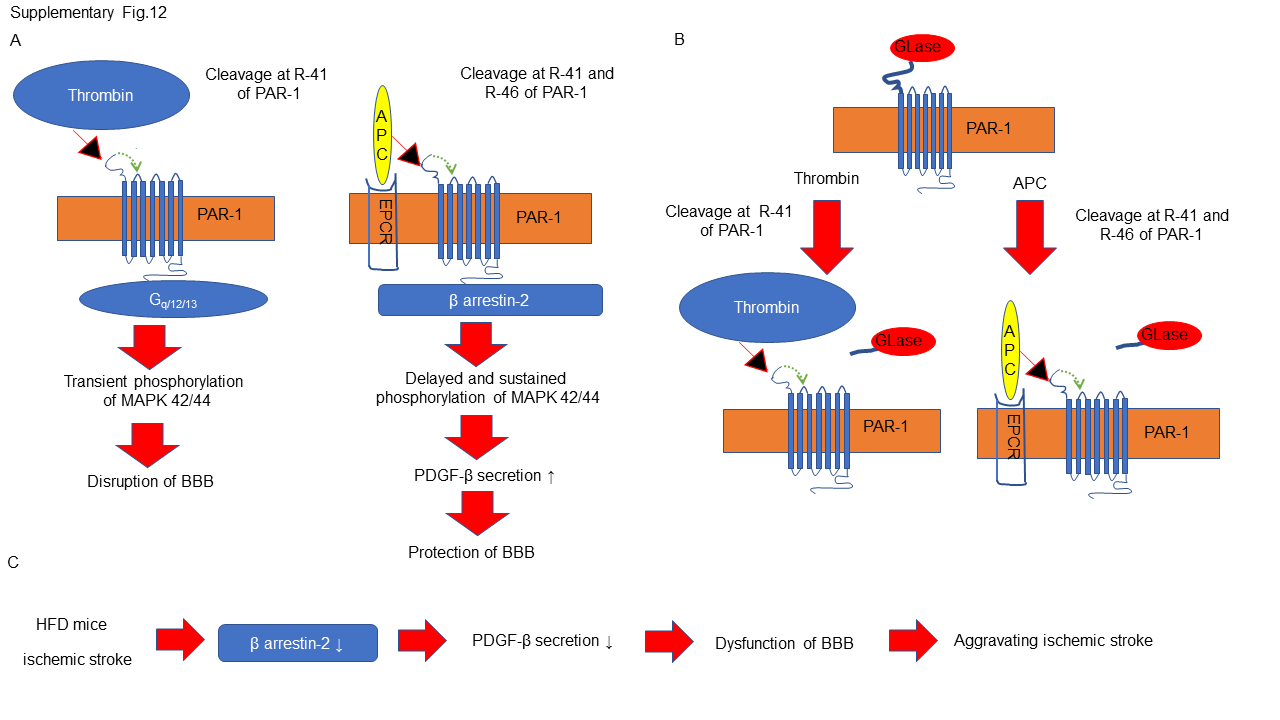

Supplement: Supplementary file 12 — Supplementary Figure.12 [file 41419_2019_1375_MOESM12_ESM.tif]
