# Supplementary material for: β-arrestin-2 in PAR-1-biased signaling has a crucial role in endothelial function via PDGF-β in stroke
Source: Cell Death Dis. 2019 Feb 4;10(2):100. doi: 10.1038/s41419-019-1375-x (PMC6361911; doi:10.1038/s41419-019-1375-x)
Supplement: Supplementary file 13 — supplemental figure legends [file 41419_2019_1375_MOESM13_ESM.docx]

**Supplementary Figure Legends**

**Supplementary Figure 1. Knockdown of β arrestin-2 by miRNA (miR β arrestin-2) shows less migration under treatment APC.**

(A) The expression level of β arresitn-2 at β arrestin-2 knockdown cells (miR-β arrestin-2) or overexpression of β arrestin-2 cells compared with transfecting control miRNA HUVEC cells (miR Cont) was shown. (B) Relative migration ratio was shown compared with control. Results are means ± SEM (n = 6). *p < 0.05 compared with control under no treatment by Mann-Whitney U tests. NS meant not significantly changed by Mann-Whitney U tests.

**Supplementary Figure 2.** **The temporal profiles of MAPK 42/44 phosphorylation by APC (3 µg/mL).**

(A) This panel showed western blot analysis of phosphorylation of MAPK 42/44 under treatment with APC 3 µg/mL. (B) Quantitative results of western blot analysis of the treatment with APC. Results are means ± SEM (n = 6). *p < 0.05 compared with the HUVECs under no treatment by Mann-Whitney U tests.

**Supplementary Figure 3.** **Knockdown of β arrestin-2 leads to less activity of MAPK 42/44.**

This panel showed western blot analysis of phosphorylation of MAPK 42/44, MPAK 42/44, PDGF-β and β-actin using β arrestin-2 knock down cells (miR β arrestin-2) compared with control cells (miR Cont) under ischemic condition.

**Supplementary Figure 4. Construction of the expression vector for GLase-PAR1.**

To express GLase-PAR1 (the fusion protein of wild-type PAR1) under the control of the cytomegalovirus (CMV) promoter, a vector of pcDNA3-PAR1sp-pGLuc-PAR1 was constructed. (A) Schematic representation of amino acid sequence of GLase-PAR1 protein expressed with pcDNA3-PAR1sp-pGLuc-PAR1. *PAR1sp*, the human PAR-1 cDNA corresponds to signal peptide sequence for secretion + 3aa (PAR1sp, M1–A26 + R27–P29 of human PAR-1 protein); *pGLuc*, a preferred human codon optimized gene of *Gaussia* luciferase (GLase, K18-D185) without its signal peptide sequence; *PAR1*, the human PAR-1 cDNA corresponds mature PAR-1 protein (PAR1, R27–T425). R41 and R46 of human PAR-1 are red-colored and bolded. (B) Schematic representation of vector construction to express GLase-PAR1. As a basic vector to express a protein of interest fused to the N- and C-terminus of GLase (without its signal peptide sequence), pcDNA3-pGLuc-pNC was constructed as follows: The *Eco*RI-*Xho*I fragment of pGLuc (*1) was obtained by polymerase chain reaction (PCR) using a primer set of pGLuc-F1^1^ and pGLuc-R2 (Supplementary Fig. 4 C), and pcDNA3-pGLuc^2^ as a template. The PCR product was digested with *Eco*RI and *Xho*I and then inserted into pcDNA3-pNC^1^ to obtain pcDNA3-pGLuc-pNC. In the multi-cloning site of pcDNA3-pGLuc-pNC, rare human codons (colored red: <http://www.kazusa.or.jp/codon/)> were not contained (codon usage frequency per 1,000 bases is shown over each codon). To obtain pcDNA3-PAR1sp-pGLuc-PAR1, pcDNA3-PAR1sp-pGLuc-pC was constructed first. The *Hind*III-*Eco*RI fragment of *PAR1sp* (*2) was obtained by PCR using a primer set of PAR1sp-F1 and PAR1sp-R1 (Supplementary Fig. 4 C) from PAR-1 cDNA IMAGE clone (3343051; Dharmacon). The PCR product was digested with *Hind*III and *Eco*RI, and then inserted into pcDNA3-pGLuc-pNC to obtain pcDNA3-PAR1sp-pGLuc-pC. Next, the *Xho*I-*Xba*I fragment of *PAR1* (*3) was obtained by PCR using a primer set of PAR1-F1 and PAR1-R1 (Supplementary Fig. 4 C) from the human PAR-1 cDNA clone, and the PCR product was digested with *Xho*I and *Xba*I, and then inserted into pcDNA3-PAR1sp-pGLuc-pC to obtain pcDNA3-PAR1sp-pGLuc-PAR1. (C) PCR primers used for vector construction.

**Supplementary Figure 5. Luminescence activities of the culture medium of HEK293 cells transiently expressing GLase-PAR1 determined by a luminometer.**

HEK293 cells (1 × 10^5^ cells) cultured on a collagen-coated 24-well plate and transiently expressing GLase-PAR1 (R41/R46 wild-type of human PAR-1) with or without EPCR were used. (A) Cells expressing GLase-PAR1 with or without EPCR were incubated for 1 h with APC (3 µg/mL) or thrombin (0.5 U/mL). (B) Cells expressing both GLase-PAR1 and EPCR were incubated for 1 h with APC at indicated concentrations. Luminescence activities in the culture medium were determined by a Atto AB-2200 luminometer. The increase in GLase activity by APC was a dose-dependent (0.3-10 µg/mL). Each figure was representative of several experiments in duplicate.

**Supplementary Figure. 6. Luminescence activities of the culture medium of endothelial bEnd.3 cells transiently expressing GLase-PAR1 determined by a luminometer.**

Endothelial bEnd.3 cells (5 × 10^4^ cells) cultured on a collagen-coated 12-well plate and transiently expressing both GLase-PAR1 (R41/R46 wild-type of human PAR-1) and EPCR were used. Cells were incubated for 0-60 min with 0.05 U/mL thrombin (A) or 3 µg/mL APC (B). Luminescence activities in the culture medium were determined by a Atto AB-2200 luminometer. Each figure was representative of several experiments in duplicate.

**Supplementary Figure 7. Construction of the expression vector for PDGF-β-GLase.**

To express the fused protein of human PDGF-β to the amino terminus of GLase (PDGF-β-GLase), under the control of the cytomegalovirus (CMV) promoter, a vector of pcDNA3- PDGF-β-pGLuc was constructed. (A) The nucleotide sequence of a synthetic gene of human PDGF-β preproprotein (*PDGF-β*). (B) Schematic representation of vector construction to express PDGF-β-GLase. To express PDGF-β-GLase, pcDNA3- PDGF-β-pGLuc was constructed as follows. The *Hin*dIII-*Eco*RI fragment of *PDGF-β* (*1) was obtained by PCR using a primer set of PDGFb-F1 and PDGFb-R1 (Supplementary Fig. 7 C) from the synthetic gene. The PCR product was digested with *Hin*dIII and *Eco*RI and then inserted into pcDNA3-pGLuc-pN^1^ to obtain pcDNA3- PDGF-β-pGLuc. (C) PCR primers used for vector construction.

**Supplementary Figure 8. Luminescence activities of the culture medium and cell lysate of endothelial bEnd.3 cells transiently expressing PDGF-β-GLase determined by a luminometer.**

(A) Schematic representation of the fusion protein of PDGF-β and GLase (PDGF-β-GLase). To express PDGF-β-GLase, the pcDNA3- PDGFβ-pGLuc expression vector was used. The hatched region corresponds the mature peptide. (B) Endothelial bEnd.3 cells (3 × 10^5^ cells) were cultured on a collagen-coated 24-well plate and transiently transfected for 72 h with the expression vector of PDGF-β-GLase and adeno-miRNA for β-arrestin-2 (or a control miRNA). Luminescence activities in the culture medium and the cell lysate were determined by a Centro XS3 LB960 luminometer. Luminescence activity per well was expressed as the mean values ± S.D. (n = 8, **p* < 0.05 compared with multiple groups by Kruskal-Wallis tests). (C) Schematic representation of GLase with its signal peptide (GLsp). To express GLsp-GLase, pcDNA3-pGLuc^2^ was used. (D) β arrestin-2 miRNA did not inhibit the luminescence activity of GLase with its signal peptide sequence (GLsp-GLase) secreted into the culture medium. Luminescence activity per well was expressed as the mean values ± S.D. (n = 8).

**Supplementary Figure.9 MEK inhibitor suppresses APC-induced PDGF-β upregulation.**

Results of quantitative RT-PCR of PDGF-β under treatment of APC were shown compare with control HUVECs. MEK inhibitor U0126 (10 µM) or PD98059 (10 µM) were preincubated for 30 min, respectively. Results are means ± SEM (n= 4 to 6). *p < 0.05 compared with multiple groups by Kruskal-Wallis tests.

**Supplementary Figure 10. HFD mice shows more BBB breakdown.**

This panel showed Evans Blue leakage of NCD mice and HFD mice (NCD, 0.65 ± 0.1 /g, n = 4; HFD, 2.9 ± 0.7 /g, n = 4; p < 0.05). Results are means ± SEM. *p < 0.05 compared with NCD mice by Mann-Whitney U tests.

**Supplementary Figure 11.** ***In vitro* HFD model, low expression level of β　arrestin-2 is detected.**

(A) This panel showed western blot analysis of β arrestin-2 under treatment of palmitic acid conjugated with 10 % BSA compared with under treatment of control (EtOH) at HUVECs. (B) Quantitative results of western blot analysis of the treatment with palmitic acid. Results are means ± SEM (n = 5). *p < 0.05 compared with control (EtOH) by Mann-Whitney U tests.

**Supplementary Figure 12. APC/EPCR complex interacts with PAR-1.**

(A) Left panel shows the signaling under thrombin. Thrombin cleavages at Arg 41 of PAR-1 and PAR-1 is activated, subsequently G_q_ and G_12/13_ are activated, and then phosphorylated MAPK 42/44 transiently, resulting in a barrier disruptive reaction. Right panel shows the signaling of PAR-1 activation by APC. APC in complex with EPCR cleavages at Arg 41 and Arg 46 of PAR-1 and preferentially triggers β arrestin-2 dependent signaling pathways. These β arrestin-2 dependent signal transduction lead to delayed and sustained phosphorylation of MAPK 42/44, and then secretion of PDGF-β, resulting in a barrier protective response. (B) The dynamic of GLase-PAR1 under treatment with thrombin or APC is shown. GLase-PAR1 expressed on the surface of living cells is cleaved by thrombin or APC in complex with EPCR, GLase is released from the cells into the culture medium. (C) In stroke or HFD-induced obesity, the β arrestin-2 dependent signaling pathways related to barrier protection are mitigated, resulting in worsening ischemic stroke and defective neurological functions.

References:

1. Yokawa, S. et al. Visualization of glucagon secretion from pancreatic alpha cells by bioluminescence video microscopy: Identification of secretion sites in the intercellular contact regions. *Biochem Biophys Res Commun* **485(4),** 725-730 (2017).

2. Inouye, S., Sahara-Miura, Y., Sato, J. & Suzuki, T. Codon optimization of genes for efficient protein expression in mammalian cells by selection of only preferred human codons. *Protein Expr Purif* **109,** 47-54 (2015).
